# Advances in Immunotherapy for Intrahepatic Cholangiocarcinoma

**DOI:** 10.3390/ijms27146228

**Published:** 2026-07-13

**Authors:** Huimin Qi, Jialin Pan, Hailong Wu

**Affiliations:** 1 Graduate School, Shanghai University of Traditional Chinese Medicine, Shanghai 201203, China; 2 Collaborative Research Center for Biomedicines, Shanghai University of Medicine and Health Sciences, Shanghai 201318, China; 3 Shanghai Key Laboratory of Molecular Imaging and Digital and Intelligent Empowerment Biomedical Innovation Center, Shanghai University of Medicine and Health Sciences, Shanghai 201318, China

**Keywords:** intrahepatic cholangiocarcinoma, tumor immune microenvironment, immunotherapy, combination therapy

## Abstract

Intrahepatic cholangiocarcinoma (iCCA) is a highly lethal and heterogeneous primary liver malignancy with a dismal prognosis. Approximately 70% of patients are diagnosed at locally advanced or metastatic stages, therefore missing the opportunity for curative surgery, and conventional chemotherapy offers limited survival benefits. Immunotherapy, especially immune checkpoint blockade, represents a promising strategy, yet its efficacy as monotherapy in iCCA remains modest primarily due to the profoundly immunosuppressive and desmoplastic tumor microenvironment. This review examines the immune cell infiltration landscape of iCCA, focusing on the distinct roles of lymphoid cells and myeloid cells in shaping immune evasion. We then analyze key factors affecting immune responses, such as tumor-intrinsic driver mutations, immune regulatory mechanisms, and acquired resistance. Furthermore, we summarize current clinical advances in iCCA immunotherapy, including immune checkpoint inhibitor monotherapy, bispecific antibodies, combination strategies with chemotherapy or targeted therapy, cancer vaccines, and adoptive cell therapy. Despite some progress, the overall response to immunotherapy remains suboptimal, and future strategies need to focus on deciphering context-specific resistance mechanisms and enhancing the tumor-specific immune response.

## 1. Introduction

Intrahepatic cholangiocarcinoma is a malignant tumor originating from intrahepatic biliary epithelial cells lining the small and large bile ducts. It is the second most prevalent primary liver cancer following hepatocellular carcinoma and is associated with a poor prognosis. Histologically, conventional iCCA can be further divided into small-duct and large-duct types [1,2,3]. Small-duct iCCA usually arises in the peripheral liver, often presenting as a mass-forming tumor [4]. In contrast, large-duct iCCA usually originates in large intrahepatic bile ducts or the perihilar region, and often shows mucin-producing glandular structures, periductal-infiltrating growth, perineural or lymphovascular invasion, and prominent desmoplastic stroma [1]. This desmoplastic stroma is mainly composed of cancer-associated fibroblasts, extracellular matrix components, endothelial cells, and infiltrating immune cells, and it represents an important structural and functional feature of iCCA. Rather than serving only as a passive scaffold, the dense stromal compartment can restrict cytotoxic T-cell infiltration, promote immune exclusion, and contribute to TGF-β-related immunosuppression [5,6]. In addition to histological heterogeneity, iCCA also exhibits substantial molecular heterogeneity. Most iCCAs are considered to have a relatively low baseline tumor mutational burden; however, a small molecularly defined subset shows high tumor mutational burden or microsatellite instability-high/mismatch repair-deficient status. Recent data suggest that MSI-high and TMB-high iCCA are uncommon, accounting for approximately 1.3–1.8% and 3.7% of cases, respectively [7,8]. In a recent retrospective cohort of 180 patients with biliary tract cancer, DDR mutations were detected in 28.3% of the overall cohort and in 30.0% of iCCA cases, based on 24 DDR-positive cases among 80 patients with iCCA [9]. These uncommon molecular features may have implications for platinum sensitivity, DNA damage response-targeted strategies, and immune checkpoint blockade.

**Figure 1:**
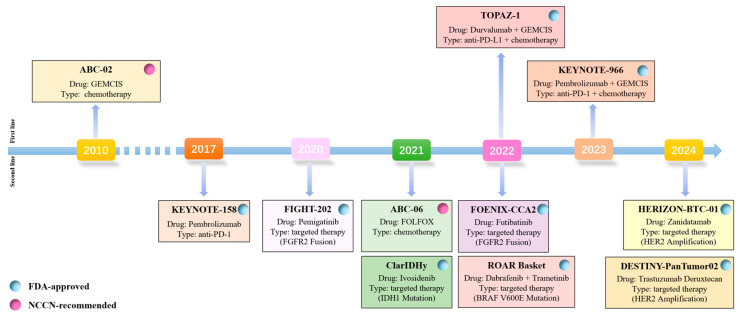
Timeline of systemic therapy landmarks in intrahepatic cholangiocarcinoma. This figure illustrates the evolution of treatment standards from 2010 to 2024, marking the transition from cytotoxic chemotherapy to targeted agents and immunotherapy. Key milestones include the establishment of gemcitabine plus cisplatin as the historical backbone, the subsequent approval of biomarker-directed therapies for *FGFR2*, *IDH1*, and *BRAF V600E* alterations, and the recent integration of immune checkpoint inhibitors into first-line regimens. The timeline highlights the shift toward a biomarker-driven treatment paradigm in iCCA management. Abbreviations: GEMCIS, gemcitabine and cisplatin; PD-L1, programmed death-ligand 1; FGFR2, fibroblast growth factor receptor 2; FOLFOX, folinic acid (Leucovorin), 5-Fluorouracil and oxaliplatin; IDH1: isocitrate dehydrogenase 1; BRAF V600E: B-Raf proto-oncogene, serine/threonine kinase (Valine-to-Glutamic acid substitution at codon 600); HER2: human epidermal growth factor receptor 2.

The epidemiology of iCCA varies significantly across different regions worldwide, but its overall incidence has been increasing annually, with the highest rates observed in East Asia and Southeast Asia [10,11]. Moreover, the mortality rate of iCCA worldwide is also showing an upward trend [11]. Notably, iCCA has an insidious onset with no specific early symptoms or reliable screening biomarkers. Most patients are already at a locally advanced stage or have developed distant metastasis at the time of diagnosis, losing the chance for curative surgery. Even after radical resection, the 5-year recurrence rate remains as high as 60–70%, and the 5-year overall survival is only 20–30% [12,13]. For advanced iCCA, gemcitabine plus cisplatin has long been the first-line standard treatment, but its efficacy is limited, with a median overall survival of less than one year, accompanied by significant toxic side effects [14]. In recent years, immunotherapy has achieved breakthrough progress in various solid tumors [15]. Treatment strategies represented by immune checkpoint inhibitors reactivate anti-tumor immune responses and bring new hope for iCCA patients. As shown in Figure 1, the treatment standards for iCCA have undergone profound changes over the past decade, evolving from simple chemotherapy to precision therapy and immunotherapy, providing new treatment options. This review starts with the immune infiltration characteristics of iCCA, systematically analyzes the key factors affecting immune responses, further reviews the current clinical progress of immunotherapy for iCCA, and critically discusses existing challenges and future research directions.

## 2. Literature Search Strategy and Study Selection

This narrative review was based on a structured literature search and was not intended to be a systematic review or scoping review. No meta-analysis was performed. The relevant literature was searched in PubMed, Web of Science, and Embase from database inception to June 2026. Additional information on ongoing or completed clinical trials was obtained from ClinicalTrials.gov, and relevant guideline or regulatory information was cross-checked when appropriate.

The search strategy combined disease-related terms, including “intrahepatic cholangiocarcinoma,” “iCCA,” “cholangiocarcinoma,” and “biliary tract cancer,” with immunotherapy- and tumor microenvironment-related terms, including “immunotherapy,” “tumor immune microenvironment,” “immune checkpoint inhibitor,” “PD-1,” “PD-L1,” “CTLA-4,” “T cell,” “B cell,” “Treg,” “NK cell,” “macrophage,” “myeloid-derived suppressor cell,” “dendritic cell,” “cancer vaccine,” “bispecific antibody,” “adoptive cell therapy,” and “clinical trial.”

Studies were considered relevant if they focused on iCCA or provided clinically meaningful iCCA subgroup data related to the tumor immune microenvironment, immune regulatory mechanisms, immunotherapy strategies, or clinical trial outcomes. Peer-reviewed original studies, clinical trials, translational studies, authoritative guidelines, and highly relevant recent reviews were prioritized. Studies focusing exclusively on non-iCCA biliary tract cancers were included only when iCCA-specific evidence was limited and the findings were relevant to iCCA. Non-English articles, duplicate reports, conference abstracts without sufficient details, and studies with limited relevance to iCCA immunotherapy were excluded.

Because this review is narrative in nature, a formal risk-of-bias assessment or evidence grading system was not applied. Nevertheless, the relevance and strength of evidence were considered during manuscript preparation, with greater emphasis placed on iCCA-specific studies, prospective clinical trials, large genomic or immunohistochemical cohorts, and mechanistically informative translational studies.

## 3. Immune Cell Infiltration Characteristics of Intrahepatic Cholangiocarcinoma

**Figure 2:**
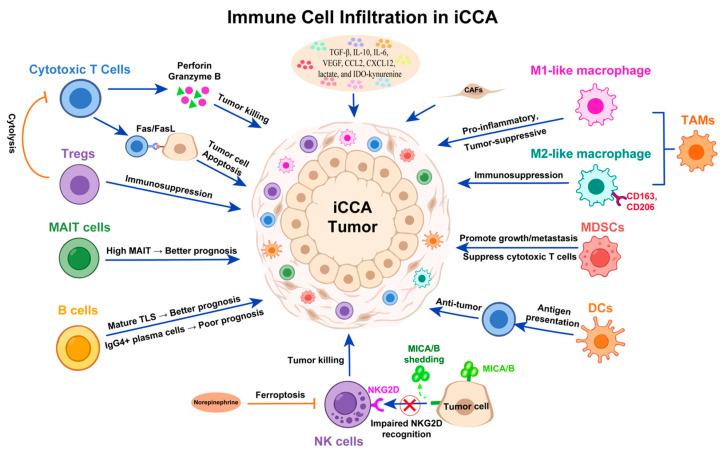
Immune Cell Infiltration in intrahepatic cholangiocarcinoma. This figure illustrates the major immune cell populations within the tumor microenvironment of iCCA and their roles in antitumor immunity and immunosuppression. The blue lines indicate different effects or influences on tumor cells or immune cells, whereas the orange lines indicate the induction of cell death through mechanisms such as apoptosis and ferroptosis. Cytotoxic T cells and NK cells contribute to tumor cell killing, DCs mediate antigen presentation, Tregs and MDSCs promote immunosuppression, TAMs exhibit both pro- and anti-tumor functions depending on their subtype, and B cells and MAIT cells can influence prognosis. Collectively, this schematic highlights the coexistence of immune activation and immunosuppressive mechanisms in the iCCA tumor microenvironment. Abbreviations: iCCA, intrahepatic cholangiocarcinoma; Tregs, regulatory T cells; MAIT cells, mucosal-associated invariant T cells; TLS, tertiary lymphoid structures; NK cells, natural killer cells; NKG2D, natural killer group 2 member D; MICA/B, MHC class I chain-related proteins A and B; TAMs, tumor-associated macrophages; MDSCs, myeloid-derived suppressor cells; DCs, dendritic cells; CD163, cluster of differentiation 163; CD206, cluster of differentiation 206; Fas/FasL, Fas receptor/Fas ligand; TGF-β, transforming growth factor-beta; IL-10, interleukin-10; IL-6, interleukin-6; VEGF, vascular endothelial growth factor; CCL2, C-C motif chemokine ligand 2; CXCL12, C-X-C motif chemokine ligand 12; lactate, lactic acid; IDO-kynurenine, indoleamine 2,3-dioxygenase-kynurenine pathway.

The tumor microenvironment of intrahepatic cholangiocarcinoma is characterized by extreme inter- and intra-tumoral heterogeneity and profound immunosuppressive features. The overall infiltration level of immune cells is relatively low, with most immune cells retained in the tumor stroma or at the invasive margin, while sparse infiltration is observed within the tumor parenchyma. According to cell lineage, the infiltrating immune cells can be mainly divided into lymphoid cells and myeloid cells (Figure 2).

### 3.1. Lymphoid Cells

T cells are the most abundant lymphocyte population in iCCA. CD8^+^ cytotoxic T lymphocytes (CTLs) are the core effectors of anti-tumor immunity. They recognize and directly kill tumor cells by releasing perforin and granzyme B, as well as through the Fas/FasL-mediated cell death pathway. Unfortunately, as the key anti-tumor effector cells, CD8^+^ cytotoxic T cells show sparse infiltration in most iCCA cases. A previous study by Sylvie Job showed that only about 11% of patient tumor samples exhibited an inflammatory subtype with T-cell infiltration within the tumor parenchyma [16]. Even worse, regulatory T cells (Tregs), a lymphocyte population associated with immunosuppressive status and poor response to immunotherapy, are often enriched in the tumor microenvironment of iCCA and confer iCCA tumors with stronger immunosuppressive characteristics [17]. Single-cell transcriptome sequencing further revealed that Tregs infiltrating iCCA display a highly activated state and specifically express the transcription factor MEOX1, whose expression level is closely associated with poor patient prognosis. Mucosal-associated invariant T (MAIT) cells are a subset of innate-like T cell that are predominantly found in the liver. In iCCA, the number of MAIT cells in tumor tissue is significantly reduced compared with adjacent non-tumor liver tissue. Christine L. et al. used Vα7.2 as a marker for MAIT cells and performed IHC staining on 48 nontumor tissues and 44 tumor tissues, showing that MAIT cell levels in tumor tissues were significantly lower than those in nontumor tissues. In this study, representative flow cytometry analysis revealed that MAIT cells accounted for 21.8% in the surrounding non-tumor liver tissue and 5.3% in the tumor. High levels of MAIT cell infiltration are independently associated with long-term patient survival [18].

B-cell infiltration in iCCA is relatively rare. Tumor-infiltrating B cells often exhibit an immature phenotype, with reduced effector function and enhanced immunosuppressive features [19]. In a small number of cases, mature tertiary lymphoid structures (TLSs) composed of CD20^+^ B cells, plasma cells, and follicular dendritic cells can form, and their presence is associated with a better prognosis [20]. Furthermore, infiltration of IgG4^+^ plasma cells is independently associated with poor prognosis, and their ratio to CD8^+^ T cells can serve as a prognostic stratification indicator. The IgG4^−^/CD8^+^ group has the best prognosis, while the IgG4^+^/CD8^−^ group has the worst outcome [21].

Natural killer cells are also relatively few in number within iCCA tumors. Infiltrating NK cells often show downregulation of activating receptors, such as NKG2D, NKp30, and NKp46 [22,23]. High expression of the chemokine CXCL9 promotes NK cell recruitment into the tumor and is associated with prolonged postoperative survival of patients [24]. In iCCA, increased shedding of MICA/B generates soluble MICA/B, which attenuates NKG2D-mediated recognition and impairs NK cell function [23]. Additionally, in iCCA with perineural invasion, norepinephrine can induce NK cell ferroptosis via the ADRB2 signaling pathway [25]. These findings suggest that targeting MICA/B shedding or blocking the ADRB2-mediated ferroptosis pathway may restore the anti-tumor function of NK cells and provide new combination strategies for iCCA immunotherapy.

### 3.2. Myeloid Cells

Myeloid cells are the most abundant and functionally complex immune population in the iCCA tumor microenvironment. They mainly include tumor-associated macrophages (TAMs), myeloid-derived suppressor cells (MDSCs), dendritic cells (DCs), and other granulocytes. These cells play critical roles in tumor progression and immune evasion.

Tumor-associated macrophages play important regulatory roles in the immune microenvironment of intrahepatic cholangiocarcinoma. Immunohistochemistry and mass cytometry analyses have shown that CD68^+^ macrophages are the most abundant myeloid population in the iCCA microenvironment. Most of them co-express CD163 and CD206, displaying a typical M2-like polarized state and tending to suppress anti-tumor immune responses [22,26]. These cells are widely distributed in the tumor parenchyma, stroma, and necrotic areas, with the highest density in hypoxic regions and at the invasive front. In contrast, M1-like macrophages are generally characterized by pro-inflammatory and potentially anti-tumor functions, including the production of nitric oxide, reactive oxygen species, and inflammatory cytokines, as well as the promotion of cytotoxic immune responses [27]. However, compared with M2-like TAMs, the distribution, functional state, and clinical significance of M1-like macrophages in iCCA remain relatively understudied and warrant further investigation. The infiltration pattern of macrophages differs among histological subtypes of iCCA: the large-duct type tends to have more abundant macrophage aggregation, whereas the small-duct type has relatively less [28]. Laurence et al. showed in a mouse model of iCCA that stimulating immune cells such as macrophages and dendritic cells through the CD40 receptor can enhance the efficacy of anti-PD-1 immunotherapy [29]. Chen et al. demonstrated that TAMs are reprogrammed under the influence of tunnel nanotube (TNT)-mediated tumor-derived arachidonic acid, exhibiting immunosuppressive characteristics [30]. In addition, the protein trefoil factor 3 can also induce M2 polarization of TAMs, thereby promoting tumor progression [31]. A recent study showed that TAMs in iCCA can induce immunosuppression by expressing MARCO, thereby promoting iCCA progression [32]. Edward et al. used multiple mouse models and patient samples to show that DKK1 promotes immune evasion in iCCA by recruiting M2 immunosuppressive macrophages [33]. These studies provide new ideas and targets for immunotherapy of iCCA.

Myeloid-derived suppressor cells are a group of immature cells derived from the myeloid lineage that functionally suppress cytotoxic T cells and promote tumor growth, invasion, and metastasis [34]. The compensatory response of MDSCs is an important mechanism of immunotherapy resistance. Studies have shown that targeting TAMs alone in iCCA leads to compensatory accumulation of granulocytic MDSCs (G-MDSCs), resulting in treatment resistance. This suggests that combination immunotherapy targeting both TAMs and G-MDSCs may be a promising strategy [35].

Dendritic cells are the key antigen-presenting cells in the tumor immune microenvironment. They capture tumor antigens and present them to T cells, resulting in activation of T-cell-mediated anti-tumor immune responses. The abundance of DCs is closely related to patient prognosis. High density of DCs is generally associated with greater CD8^+^ T-cell infiltration and better long-term survival, whereas low DCs abundance is associated with lymph node metastasis (LNM) and poor prognosis in iCCA. Conducting scRNA-seq and bulk RNA-seq analyses on 14 iCCA samples (five with LNM, nine without LNM), Sun et al. demonstrated that patients with LNM exhibit significantly reduced DC infiltration into primary tumor sites compared to those without LNM [36]. Conventional dendritic cells are divided into conventional type 1 DCs (cDC1s) and conventional type 2 DCs (cDC2), which have different functions. cDC1 is mainly responsible for antigen presentation. It presents antigens to CD8^+^ T cells via MHC class I molecules, thereby activating cytotoxic T cells and playing an important role in anti-tumor immunity. cDC2 mainly presents antigens to CD4^+^ T cells via MHC class II molecules [37]. Pei et al. showed in a mouse model of iCCA that the infiltration rate of cDC1 is significantly lower in advanced iCCAs compared with early-stage iCCAs. Expanding and activating CD103^+^ cDC1 via combination of Flt3L and poly I:C (FL-pIC therapy) significantly suppressed tumorigenesis in AKT/YAP-driven mouse iCCA and made the tumors more sensitive to anti-PD-1 therapy [38]. The infiltration level and functional status of dendritic cells directly affect antigen presentation, effector T-cell activation, and immunotherapy response, making them potential targets for optimizing immunotherapy in the future.

In summary, myeloid cells play complex immunomodulatory roles in the immune microenvironment of iCCA. They not only influence tumor immune evasion but also provide potential targets for immunotherapy. A deeper understanding of the mechanisms of myeloid cells may offer new insights for immunotherapeutic strategies against iCCA.

### 3.3. Immune-Cell Interactions and Immune Phenotypes in iCCA

Collectively, immune cells in iCCA do not function as isolated populations. Instead, they interact with each other and form spatially organized immune networks. MDSCs can suppress T-cell responses by expressing PD-L1 and depleting essential amino acids, such as L-arginine and tryptophan, thereby impairing T-cell activation, proliferation, and effector function [39,40,41]. Tregs further reinforce this immunosuppressive state by suppressing effector T-cell proliferation and function through CTLA-4-mediated inhibition of costimulatory signaling, IL-10 and TGF-β secretion, and IL-2 consumption [17,34,42,43]. Furthermore, Tregs downregulate NKG2D expression on NK cells via membrane-bound TGF-β and directly suppress NK cell effector functions [44]; they also inhibit IL-2-dependent NK cell activation through high expression of the IL-2α receptor (CD25) [45]. In contrast, mature DCs promote antitumor immunity by capturing and presenting tumor antigens through MHC molecules and providing costimulatory signals to prime tumor-specific T cells [46]. B cells, follicular helper T cells, DCs, and other lymphoid populations can organize into TLSs, thereby supporting a more coordinated antitumor immune response [47]. The balance between these immune-activating and immunosuppressive interactions ultimately determines whether the iCCA immune microenvironment shifts toward antitumor activation or immune suppression.

Based on the composition, abundance, and localization of these cellular components, distinct immune phenotypes have been proposed to explain the immune heterogeneity and potential therapeutic vulnerabilities of iCCA. Lin et al. divided 255 iCCA cases into three immune subgroups: an immune-suppressive subgroup enriched in neutrophils and immature dendritic cells; an immune-exclusion subgroup associated with high tumor purity, HLA loss of heterozygosity, and antigen-presentation defects; and an immune-activated subgroup enriched in adaptive immune cells, NK cells, activated dendritic cells, tumor-infiltrating lymphocytes, and tertiary lymphoid structures (TLSs) [48]. Ding et al. showed that the spatial distribution and density of TLSs in 962 iCCA cases were closely associated with prognosis, with intratumoral TLSs linked to favorable survival and peritumoral TLS enrichment associated with poorer outcomes [47]. Job et al. classified iCCA into four TME-based immune subtypes: immune-desert (48%), immunogenic (9%), myeloid-rich (13%), and mesenchymal (28%) [16]. The immune-desert subtype showed globally reduced immune and stromal signatures, minimal lymphoid or myeloid infiltration, and almost no immune cells within tumor nests, suggesting the need for immune-priming strategies to convert cold tumors into inflamed tumors. The immunogenic subtype was characterized by abundant peritumoral and intratumoral CD4^+^, CD8^+^, and memory T-cell infiltration, B-cell clusters, macrophages, MHC class I/II expression, TLS-related features, and immune-checkpoint activation, supporting potential sensitivity to immune checkpoint blockade. The myeloid-rich subtype was enriched in monocyte/macrophage and M2 macrophage signatures with limited effective adaptive immunity, suggesting potential benefit from macrophage-depleting or macrophage-reprogramming strategies. The mesenchymal subtype was dominated by activated fibroblast and hepatic stellate cell signatures, extracellular matrix remodeling, angiogenesis, and limited intratumoral immune infiltration, suggesting that stromal- or antifibrotic-targeting approaches may help overcome immune exclusion. These studies indicate that different immune phenotypes are associated with distinct mechanisms of immune dysfunction. Therefore, a better understanding of the immune-cell composition and heterogeneity of the iCCA immune microenvironment provides an important basis for elucidating immune escape and developing phenotype-guided immunotherapeutic strategies.

## 4. Factors Affecting Immune Response in Intrahepatic Cholangiocarcinoma

### 4.1. Tumor-Intrinsic Drivers

The genetic characteristics of tumor cells can influence the phenotype of the tumor immune microenvironment, thereby affecting their sensitivity to immunotherapy [49]. Although immune checkpoint inhibitors have achieved breakthroughs in many tumor types, their efficacy as monotherapy in iCCA is limited. This has prompted researchers to investigate the role of tumor-intrinsic factors in regulating immune responses. Driver gene mutations are among the core determinants of immune microenvironment heterogeneity in intrahepatic cholangiocarcinoma [43]. Common driver mutations in iCCA include *FGFR2*, *IDH1/2*, *KRAS*, *BAP1*, *ARID1A*, etc. [50]. *FGFR2* fusions are present in approximately 7–15% of iCCA cases. *FGFR2* fusions/mutations have been linked to the immune contexture of iCCA. *FGFR2* fusions/mutations have been linked to the immune contexture of iCCA, but existing studies do not consistently indicate a uniform immune-suppressive or immune-activated state, and direct mechanistic evidence remains limited. In the cohort analyzed by Lin et al., *FGFR2* fusions and mutations were associated with reduced immune-cell infiltration, including lower levels of total TILs, CD4^+^ T cells, and macrophages [51]. A CODEX multiplexed imaging study at the single-cell level in 24 iCCA cases revealed that *FGFR*2^+^ tumors display an immune desert phenotype, with significantly reduced CD8^+^ T cells. Moreover, CD4^+^ T cells were located farther from tumor cells, while CD11b^+^/CD15^+^ granulocytes were closer to tumor cells, suggesting that *FGFR* signaling may impede T-cell infiltration through a spatial exclusion mechanism [52]. However, another iCCA cohort has reported that *FGFR*2 fusion/rearrangement is associated with reduced Treg and N2-neutrophil infiltration, increased N1-neutrophil infiltration, favorable prognosis, and a potentially immune-activated state [53]. Although *FGFR* inhibitors remain the most evidence-based molecularly guided treatment option for *FGFR2* fusion/rearrangement-positive iCCA, the heterogeneous immune features observed in *FGFR2*-altered tumors suggest that *FGFR* inhibition may have potential immune-modulating effects beyond direct tumor-cell targeting [54]. *FGFR2* fusion/rearrangement has not yet been validated as a predictive biomarker for ICI benefit. This limitation may be explained by the heterogeneous immune phenotypes reported across different cohorts, the limited number of iCCA-specific studies, and the lack of prospective trials stratifying ICI efficacy by *FGFR2* status [51,52,53]. Clinically, *FGFR*2-targeted therapy plus immunotherapy is being explored, including pemigatinib plus durvalumab in *FGFR2* fusion/rearrangement-positive advanced iCCA (NCT06728410). Therefore, whether *FGFR*2-altered patients can derive additional benefit from *FGFR*-targeted therapy combined with ICIs remains investigational. *IDH1/2* mutations occur in 10–20% of iCCA cases. The mutant *IDH* enzyme converts α-ketoglutarate (α-KG) to the oncometabolite D-2-hydroxyglutarate (D-2-HG). D-2-HG inhibits lactate dehydrogenase (LDHA) activity, thereby impairing glycolysis in CD8^+^ T cells, leading to functional impairment and suppressed proliferation [55]. Furthermore, D-2-HG acts as a competitive inhibitor that blocks the activity of TET2 DNA demethylase, which is essential for tumor cells to respond to IFNγ signaling [56]. Depletion of CD8^+^ T cells or conditional knockout of TET2 in tumors results in resistance to immunotherapy [57,58]. In addition, *IDH1*-mutant tumor cells were shown to upregulate CCL2, leading to increased recruitment and polarization of M2-like TAMs, reduced CD8^+^ T-cell infiltration, and an immunosuppressive microenvironment [59]. Large-scale genomic profiling confirmed that *IDH*1/2-mutant iCCA has significantly lower tumor mutational burden (TMB), microsatellite instability-high (MSI-H) proportion, and PD-L1 expression compared with wild-type tumors. In addition, resting NK cells are reduced and the immunosuppressive molecule B7-H4 is elevated [52]. Therefore, *IDH*-mutant iCCA is considered a typical immunosuppressive tumor type. For *IDH*1-mutant iCCA, *IDH*-mutant iCCA typically shows immune-cold features, which may limit the efficacy of ICIs. Whether *IDH*1/2 mutations can help select patients for *IDH*-targeted therapy combined with immunotherapy remains uncertain, because prospective clinical evidence in iCCA is still lacking. Preclinical evidence suggests that *IDH*1 inhibition can restore IFNγ signaling and enhance CD8^+^ T-cell recruitment, thereby providing a rationale for combination with ICI or other immune-enhancing strategies. Although clinical validation is still needed, this approach represents a promising direction for genotype-guided immunotherapy in iCCA. *KRAS* mutations are also common in iCCA, with a mutation frequency of approximately 19%, and are often associated with genomic instability and an inflammatory microenvironment [50]. A study by Zhang et al. found that *KRAS* mutation is closely linked to alternative splicing of IL1RN in iCCA. Mechanistically, *KRAS* mutation activates ERK1/2 signaling, which upregulates the inflammatory cytokine CXCL3 and drives neutrophil recruitment. At the same time, the mutation promotes the expression and secretion of specific IL1RN splice variants, particularly IL1RN-201/203. In *KRAS*-mutant iCCA mice, IL1RN-201/203 suppresses neutrophil infiltration and reprograms them toward an anti-tumor phenotype, while also promoting GZMB^+^ CD8^+^ T-cell infiltration, thus inhibiting tumor progression [60]. An integrative multi-omics analysis of 255 iCCA patients stratified the patients into high-inflammation and low-inflammation subgroups. *KRAS* mutations were significantly enriched in the high-inflammation subgroup, which was characterized by excessive infiltration of neutrophils and immature dendritic cells, presenting a myeloid-inflammation-driven immunosuppressive state [48]. Mechanistically, *KRAS* mutation can activate CXCL5 expression through the NF-κB pathway, recruiting CXCR2^+^ PMN-MDSCs. At the same time, it drives the production of prostaglandin E2, forming a positive feedback loop between tumor cells and PMN-MDSCs, which potently suppresses CD8^+^ T-cell anti-tumor activity via the EP4 receptor [61].

Different driver gene mutations shape distinct immune microenvironment phenotypes through their unique epigenetic, metabolic, and signaling pathway mechanisms. Combining molecular subtyping with immune subtyping not only helps to more precisely identify patient subgroups that may benefit from different immunotherapy combination strategies, but also provides a theoretical basis for the development of novel therapeutic targets based on specific driver genes.

### 4.2. Immune Regulatory Mechanisms and Acquired Resistance

Immune regulatory mechanisms refer to the various molecular pathways and cellular interactions in the tumor microenvironment that directly or indirectly affect the functional status of immune cells. Understanding these mechanisms is crucial for improving the efficacy of immunotherapy in iCCA, because they determine how the tumor escapes host immune attack and why most patients respond poorly to immune checkpoint inhibitor (ICI) monotherapy. Section 4.2 will describe T-cell suppression, impaired antigen presentation, immunosuppression mediated by soluble factors and metabolites in the iCCA microenvironment, and the acquired resistance that emerges under therapeutic pressure.

Aberrant expression of immune checkpoint molecules is a core mechanism of immune evasion in iCCA. High PD-L1 expression is observed in about 12% of iCCA cases, and is often associated with poor prognosis [62,63]. Binding of PD-1 to PD-L1 delivers inhibitory signals to T cells, inducing T-cell anergy or apoptosis. In addition to the PD-1/PD-L1 pathway, CTLA-4 also plays an important immunosuppressive role in iCCA. CTLA-4 is a second receptor for the T-cell costimulatory ligands B7-1 (CD80) and B7-2 (CD86), and functions as a negative regulator of T-cell activation, proliferation, and effector function [64]. It is mainly expressed in T-cell compartments, particularly FoxP3^+^ regulatory T cells (Tregs) and activated conventional T cells [65]. A study of 290 iCCA patients found that the density of CTLA-4-positive tumor-infiltrating lymphocytes (TILs) was significantly higher in iCCA tumors than in adjacent liver tissue. High density of CTLA-4^+^ TILs was significantly associated with reduced overall survival and increased cumulative recurrence. CTLA-4^+^ TIL density correlated positively with FOXP3^+^ regulatory T-cell density, suggesting a synergistic effect between CTLA-4 and regulatory immune cells. Beyond Tregs and activated conventional T cells, immunohistochemical staining in the same study also revealed CTLA-4 expression on tumor cells and stromal cells in iCCA; however, CTLA-4 expression on tumor cells was not associated with prognosis. Notably, the number of CTLA-4^+^ TILs exceeded that of FOXP3^+^ Tregs, suggesting that non-Treg CTLA-4^+^ TILs may also be involved in the immunosuppressive microenvironment of iCCA. [66]. T-cell exhaustion is a direct consequence of upregulated immune checkpoint molecules. Mass cytometry analysis of the iCCA tumor immune microenvironment revealed that, compared with the adjacent normal liver tissues, tumor tissues contain a substantial population of exhausted CD8^+^ T cells co-expressing CD39 and PD-1, as well as immunosuppressive regulatory T cells with high CD39 expression [26]. Moreover, the immune checkpoint regulatory network is not limited to PD-1/PD-L1 and CTLA-4. Various emerging inhibitory receptors such as LAG-3, TIM-3, TIGIT, and B7-H3 have been shown to exert suppressive effects in many tumors [67,68]. The relationships between their expression patterns, T-cell exhaustion, immune evasion, and response to immunotherapy are becoming important focuses in the development of immunotherapeutic strategies. However, the research on these receptors in iCCA is still limited and requires further exploration. A deeper understanding of how these pathways relate to immunotherapy response will help improve immunotherapeutic approaches and overcome acquired resistance.

Defective antigen presentation is another important mechanism of immune evasion in iCCA. Major histocompatibility complex class I (MHC-I) molecules are often downregulated or lost on iCCA cells, preventing CD8^+^ T cells from effectively recognizing tumor neoantigens. In a large immunohistochemical study of biliary tract cancers that included an intrahepatic cholangiocarcinoma subgroup, Goeppert et al. reported low MHC-I expression in 58.2% of intrahepatic cholangiocarcinomas [69]. An analysis of 27 iCCA patients found that although all tumors had lymphocyte infiltration, the expression levels of PD-L1 and HLA class I antigens varied among patients. HLA class I antigen expression was positively associated with CD8^+^ T-cell infiltration, and the combination of HLA class I positivity with PD-L1 negativity was related to better therapeutic responses [70]. In contrast, when HLA class I antigens were absent and PD-L1 was expressed, the tumor could effectively evade immune attack. These findings suggest that tumor-cell MHC-I/HLA-I expression is closely associated with CD8^+^ T-cell infiltration and cytotoxic immune recognition in iCCA. Reduced MHC-I expression may impair tumor antigen presentation to CD8^+^ T cells, thereby contributing to cytotoxic T-cell exclusion or ineffective antitumor responses. By contrast, the relationship between tumor-cell MHC-I expression and NK-cell infiltration in iCCA remains less clearly defined. Loss of HLA class I antigens can be caused by B2M gene mutations, repression of the transcriptional regulator NLRC5, or hypermethylation of the promoter regions of the classical HLA class I genes HLA-A, HLA-B and HLA-C, leading to impaired MHC class I expression and tumor immune evasion [71,72,73,74]. In iCCA, *IDH*1/2 mutations can lead to DNA hypermethylation, which silences antigen processing and presentation-related genes such as HLA genes, further reinforcing the immunosuppressive microenvironment [75]. In addition to MHC-I, MHC-II molecules are also heterogeneously expressed in iCCA. MHC-II molecules, represented by HLA-DR, present antigens to CD4^+^ T cells and may contribute to antitumor immune recognition when expressed by tumor cells. In an early immunohistochemical study of 20 intrahepatic cholangiocarcinomas, tumor-cell HLA-DR positivity was detected in seven cases, suggesting that absent HLA-DR expression may occur in a substantial subset of iCCA cases [76]. A single-cell RNA sequencing study showed that high-stemness malignant cells in iCCA expressed significantly lower levels of MHC-II molecules than low-stemness malignant cells [77]. This finding suggests that MHC-II downregulation may be associated with the immune-evasive phenotype of stem-like malignant cells in iCCA.

Soluble immunosuppressive factors and metabolites also play key roles in iCCA immune evasion. Transforming growth factor-β (TGF-β) is one of the immunomodulatory cytokines in the iCCA microenvironment; it is mainly secreted by cancer-associated fibroblasts (CAFs) and tumor cells. The TGF-β signaling pathway is highly active during iCCA progression, which results in enhanced tumor cell proliferation, migration, and invasion [78]. An immunohistochemical analysis of 78 iCCA cases showed that TGF-β expression was significantly associated with poor prognosis [79]. Liu et al. developed a TGF-β-associated miRNA signature (TAMIS) based on the expression of TGF-β-derived miRNAs in iCCA. This signature stratified patients into high- and low-risk groups, with the high-TAMIS group showing poorer overall and relapse-free survival. Immunologically, high-TAMIS tumors exhibited lower CD8^+^ T-cell infiltration and PD-L1 expression, suggesting a more immune-excluded or “super-cold” tumor phenotype [80]. At the metabolic level, overexpression of indoleamine-2,3-dioxygenase 1 (IDO1) is an important mechanism of immune evasion in cholangiocarcinoma. IDO is expressed in a variety of cell types within the tumor microenvironment, including tumor cells, immune cells, endothelial cells, and stromal fibroblasts, whereas its expression is generally not prominent in T cells [81]. Studies have shown that IDO1 expression is closely related to poor overall survival in cholangiocarcinoma patients, and co-expression of IDO1 and PD-L1 is associated with an even worse prognosis [82]. Mechanistically, as an intracellular metabolic enzyme, IDO1 inhibits T-cell function by consuming tryptophan and producing kynurenine [83]. Tryptophan depletion activates the GCN2 kinase in T cells, leading to eIF2α phosphorylation, inhibition of protein synthesis, cell cycle arrest, suppressed proliferation, and eventually T-cell apoptosis [84]. Meanwhile, kynurenine taken up by T cells persistently activates the aryl hydrocarbon receptor, directly suppressing CD8^+^ T-cell cytotoxicity while promoting Treg differentiation and expansion [85]. Because CD8^+^ T cells lack kynureninase to metabolize kynurenine, this immunosuppressive effect persists [86]. Furthermore, the metabolite lactate in the tumor microenvironment also has potent immunosuppressive functions. Mechanistically, tumor-derived lactate activates SREBP2-dependent mevalonate/cholesterol metabolism in dendritic cells and drives their conversion into CD63^+^ mregDCs, a tolerogenic DC population with impaired antigen cross-presentation capacity. This process suppresses CD8^+^ T-cell activation and promotes Treg differentiation, thereby weakening DC-mediated anti-tumor immune responses [87]. In summary, soluble immunosuppressive factors act synergistically through multiple mechanisms and are important contributors to immune evasion and poor immunotherapy outcomes in iCCA.

Under continuous immunotherapy pressure, iCCA can undergo adaptive changes leading to acquired resistance. Lu et al. performed single-cell sequencing on iCCA patients who received combination therapy with gemcitabine, oxaliplatin, lenvatinib, and anti-PD-1. They found that CD5L+ macrophages induced T-cell exhaustion by upregulating CTLA4 expression on CD8^+^ GZMB^+^ T cells, thereby impairing treatment response. Anti-CTLA-4 antibody reversed this resistant phenotype [88]. In addition, Tregs, TAMs, and MDSCs persist in resistant tumors and further enhance immunosuppression by secreting IL-10, TGF-β, and other factors [35].

In conclusion, iCCA achieves immune evasion through multiple immune regulatory mechanisms and evolves acquired resistance under therapeutic pressure. A deep understanding of this complex network is of great importance for the development of rational combination therapy strategies.

## 5. Clinical Advances in Immunotherapy for Intrahepatic Cholangiocarcinoma

**Figure 3:**
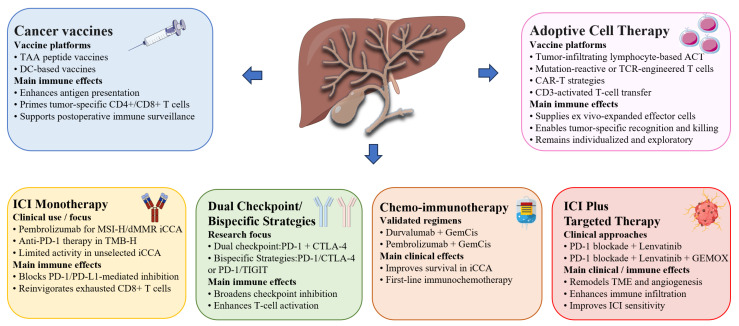
Immunotherapy strategies in intrahepatic cholangiocarcinoma. This schematic summarizes major immunotherapy-related strategies in iCCA, including ICI monotherapy, dual checkpoint blockade or bispecific antibody strategies, chemo-immunotherapy, ICI plus targeted therapy, cancer vaccines, and adoptive cell therapy. Abbreviations: ACT, adoptive cell therapy; CAR-T, chimeric antigen receptor T-cell therapy; CTLA-4, cytotoxic T-lymphocyte-associated protein 4; DC, dendritic cell; GemCis, gemcitabine plus cisplatin; GEMOX, gemcitabine plus oxaliplatin; ICI, immune checkpoint inhibitor; MSI-H/dMMR, microsatellite instability-high/mismatch repair-deficient; PD-1, programmed cell death protein 1; PD-L1, programmed death-ligand 1; TCR-T, T-cell receptor-engineered T-cell therapy; TMB-H, tumor mutational burden-high; TME, tumor microenvironment; TIGIT, T cell immune receptor with Ig and ITIM domains.

Recent breakthroughs in immunotherapy have reshaped the treatment landscape for various solid tumors, including iCCA. Although the immunosuppressive tumor microenvironment and low baseline T-cell infiltration limit the efficacy of single-agent immune checkpoint blockade, accumulating clinical evidence supports the use of combination strategies and novel immunotherapeutic modalities. As illustrated in Figure 3, current immunotherapy approaches for iCCA can be broadly categorized into three pillars: immune checkpoint inhibitors that reactivate exhausted T cells, cancer vaccines that prime tumor-specific immune responses, and adoptive cell therapies that employ genetically engineered or expanded T cells. Section 5 summarizes the clinical progress and key trials of these strategies, ranging from PD-1/PD-L1 monotherapy to bispecific antibodies, chemo-immunotherapy combinations, targeted therapy combinations, cancer vaccines, and adoptive cell transfer.

### 5.1. Immune Checkpoint Inhibitor Monotherapy

In the exploration of treatments for intrahepatic cholangiocarcinoma, the application of single immune checkpoint inhibitors is primarily based on specific molecular biological contexts. KEYNOTE-158 provided clinical evidence for pembrolizumab monotherapy in previously treated advanced biliary tract cancer (BTC), a clinically relevant disease group for iCCA. In the overall BTC cohort, 104 patients received pembrolizumab, with an objective response rate (ORR) of 5.8% (6/104), a median overall survival (OS) of 7.4 months, and a median progression-free survival (PFS) of 2.0 months [89]. Importantly, the benefit of pembrolizumab appears more pronounced in biomarker-selected MSI-H/dMMR cholangiocarcinoma, as shown in a separate MSI-H/dMMR noncolorectal cancer analysis from KEYNOTE-158. In this analysis, 22 patients with MSI-H/dMMR cholangiocarcinoma treated with pembrolizumab achieved an ORR of 40.9%, with median PFS and OS of 4.2 months and 24.3 months, respectively. Based on the tumor-agnostic efficacy of pembrolizumab in MSI-H/dMMR solid tumors, the U.S. Food and Drug Administration (FDA) approved pembrolizumab for patients with unresectable or metastatic microsatellite instability-high/mismatch repair-deficient (MSI-H/dMMR) solid tumors, including eligible patients with intrahepatic cholangiocarcinoma [90,91,92]. Therefore, pembrolizumab monotherapy represents a biomarker-driven treatment option for patients with unresectable or metastatic MSI-H/dMMR iCCA.

A multicenter phase 2 trial (NCT02829918) assessed nivolumab in 54 patients with refractory advanced biliary tract cancer, including 32 (59%) with intrahepatic cholangiocarcinoma [93]. The ORR for iCCA patients was 21% (6/28), with longer PFS observed in those expressing higher PD-L1 levels, suggesting that nivolumab provides durable clinical benefit with manageable toxicity in refractory iCCA. Notably, Olumide et al. reported a case of chemotherapy-resistant iCCA with high tumor mutational burden (TMB), with the patient achieving a sustained clinical response lasting 16 months following anti-PD-1 therapy, accompanied by resolution of disease-related symptoms [94]. This observation underscores the potential of TMB as a predictive biomarker for immune checkpoint inhibitor responsiveness. Although the overall response rate to immunotherapy remains low in cholangiocarcinoma, subsets of patients with specific molecular features may derive significant benefit. Consequently, comprehensive molecular profiling of iCCA patients is recommended to identify immunologically favorable subgroups amenable to immunotherapy [95]. This indicates that some patients can derive long-term benefit from monotherapy with immune checkpoint inhibitors.

Other PD-1/PD-L1 inhibitors such as durvalumab and atezolizumab have also been explored for monotherapy in early Phase I/II studies. Although they demonstrated durable antitumor responses in a small proportion of patients, the overall response rates remained low. Subsequent development efforts for these drugs have shifted toward combination therapy strategies [96,97,98,99,100,101,102].

### 5.2. Bispecific Antibodies and Dual Immune Checkpoint Blockade

Simultaneously blocking multiple immunosuppressive pathways is a key strategy for overcoming drug resistance. Dual immunotherapy combinations represent one research direction. Current clinical trials evaluating bispecific antibodies and dual immune checkpoint blockade are summarized in Table 1 and Table 2.

A subgroup analysis of the phase 2 nonrandomized clinical trial CA209-538 evaluated the efficacy and safety of combined nivolumab and ipilimumab immunotherapy in patients with advanced biliary tract cancer [103]. The results demonstrated an objective response rate of 31% and a disease control rate of 43.75% in the ICC cohort. This marked the first assessment of nivolumab–ipilimumab combination therapy in ICC, revealing associations with significantly improved clinical outcomes and durable treatment responses. The observed antitumor activity suggests this dual checkpoint blockade may represent a promising investigational strategy for future iCCA immunotherapy studies.

Furthermore, engineered bispecific antibodies have emerged as a prominent new focus. The international Phase II GEMINI-Hepatobiliary study is evaluating the efficacy of Volrustomig (a PD-1/CTLA-4 bispecific antibody) and Rilvegostomig (a PD-1/TIGIT bispecific antibody), each in combination with chemotherapy, as first-line treatments for biliary tract cancer. Another PD-1/CTLA-4 bispecific antibody, XmAb20717, is also under investigation in a Phase II trial (NCT05297903), focusing on patients with advanced biliary tract cancer (including iCCA) who have received prior chemotherapy but are naive to immune checkpoint inhibitor therapy. However, the development of bispecific antibodies has not been without setbacks. The PD-L1/TGF-β bifunctional fusion protein Bintrafusp Alfa encountered significant challenges. Both its Phase II and subsequent first-line Phase III trials in biliary tract cancer failed to meet their primary endpoints [104,105]. Although a trend towards improved objective response rates was observed, it did not translate into a survival benefit, and the combination therapy group exhibited an increased incidence of adverse events, notably bleeding. Several factors may explain this translational failure. Although simultaneous PD-L1 and TGF-β blockade was designed to enhance antitumor immunity, it may, in some contexts, unintentionally promote tumor progression through oncogenic signaling. Because TGF-β is involved in epithelial–mesenchymal plasticity, its inhibition in advanced or metastatic disease may not necessarily reverse immune exclusion and could instead favor metastatic tumor growth [106,107,108]. In addition, the fixed ratio of the PD-L1- and TGF-β-targeting components in Bintrafusp Alfa may have prevented simultaneous optimization of both pathways. Therefore, the Bintrafusp Alfa experience suggests that dual-target immunotherapy should be guided by a clearer understanding of tumor stage, immune phenotype, and dose optimization, rather than relying solely on theoretical pathway complementarity.

### 5.3. Immune Checkpoint Inhibitors in Combination with Chemotherapy

The combination of immune checkpoint inhibitors with standard chemotherapy represents a groundbreaking advancement in the treatment of intrahepatic cholangiocarcinoma. Pivotal phase III clinical trials, notably TOPAZ-1 and KEYNOTE-966, have validated the survival benefits of this combination strategy, thereby redefining the first-line treatment paradigm for advanced iCCA. In TOPAZ-1, 383 patients with intrahepatic cholangiocarcinoma were included in the primary tumor-location subgroup analysis, including 190 patients in the durvalumab plus GemCis group and 193 patients in the placebo plus GemCis group. In this exploratory subgroup analysis, durvalumab plus GemCis showed improved OS (13.5 vs. 11.5 months; HR, 0.76; 95% CI, 0.58–0.98), PFS (7.3 vs. 6.0 months; HR, 0.79; 95% CI, 0.64–0.99), and ORR (24.7% vs. 15.5%) in the ICC subgroup [96,109]. These findings establish durvalumab plus gemcitabine/cisplatin as the new first-line standard for advanced iCCA, providing clinically meaningful survival benefits without substantially increasing toxicity. A real-world retrospective analysis further corroborated the efficacy of durvalumab-based platinum–gemcitabine regimens [110]. Similarly, the phase 3 KEYNOTE-966 trial validated the effectiveness of pembrolizumab plus gemcitabine and cisplatin used as initial therapy for advanced cholangiocarcinoma [111,112]. In this trial, patients with an intrahepatic site of origin accounted for a substantial subgroup, including 320 patients in the pembrolizumab plus gemcitabine/cisplatin group and 313 patients in the placebo plus chemotherapy group. Site of origin was included in the prespecified subgroup analyses. In the overall BTC population, pembrolizumab plus gemcitabine/cisplatin improved OS compared with a placebo plus chemotherapy (12.7 vs. 10.9 months; HR, 0.83; 95% CI, 0.72–0.95). Consistently, the intrahepatic subgroup also showed an OS benefit (HR, 0.76; 95% CI, 0.64–0.91). These studies reinforce the central role of the “immunotherapy plus chemotherapy” model in first-line treatment for iCCA. These landmark studies have not only established immunochemotherapy as the new standard of care for advanced iCCA but also, more importantly, demonstrated that combining immune checkpoint inhibitors to conventional chemotherapy provides clinically meaningful survival improvements without substantially increasing toxicity. This therapeutic paradigm shift has laid a solid foundation for subsequent exploration of more precise combination strategies, with completed and ongoing research summarized in Table 3 and Table 4.

### 5.4. Immune Checkpoint Inhibitors in Combination with Targeted Therapy

Immune checkpoint inhibitors combined with targeted therapies offer personalized treatment options for patients with iCCA of different molecular subtypes [50,113]. Novel combinations of immunotherapy with targeted agents, with or without chemotherapy, are detailed in Table 5 and Table 6. A Phase II trial (NCT05174650) is currently underway to evaluate the efficacy of the combination therapy of the *FGFR* inhibitor derazantinib with the PD-L1 inhibitor atezolizumab in patients with advanced iCCA harboring *FGFR*2 fusions/rearrangements. This aims to establish a new treatment standard for this precisely selected patient cohort. Additionally, exploring *IDH*1 mutations represents another major focus [57,114]. This mutation leads to accumulation of the metabolite 2-hydroxyglutarate, which suppresses T-cell function [115]. IDH1 inhibitors theoretically reverse this process [116,117,118]. Accordingly, early-phase clinical trials are evaluating IDH1 inhibitor-based immunotherapy combinations, such as ivosidenib plus nivolumab (NCT04056910) in *IDH*1-mutant advanced solid tumors and ivosidenib plus nivolumab/ipilimumab (NCT05921760) in unresectable or metastatic *IDH*1-mutant cholangiocarcinoma [119].

In the field of broad-spectrum targeted therapy, strategies targeting the VEGF/VEGFR signaling pathway demonstrate unique application value. Among these, the IMbrave 151 study evaluated the combination regimen of the PD-L1 inhibitor atezolizumab with the VEGF inhibitor bevacizumab and chemotherapy [99]. In the iCCA subgroup, mPFS was 8.3 months in the atezolizumab plus bevacizumab and chemotherapy group versus 8.3 months in the atezolizumab plus chemotherapy control group, while mOS was 16.7 versus 17.2 months, respectively. These results indicate that neither the primary endpoint of PFS nor the secondary endpoint of OS was substantially improved in the iCCA subgroup. However, exploratory biomarker analysis suggested that higher VEGFA gene expression in the bevacizumab-containing arm may be associated with greater PFS benefit, indicating that VEGFA may serve as a potential predictive biomarker for selecting patients who are more likely to benefit from this combination strategy. Although it failed to meet the primary endpoint of progression-free survival, it provided important reference for optimizing this combination strategy [100]. In contrast, the combination of the multi-targeted tyrosine kinase inhibitor lenvatinib with immune checkpoint inhibitors demonstrated more pronounced clinical benefits. The randomized Phase II study NCT04361331 confirmed that first-line treatment with lenvatinib plus the PD-1 inhibitor toripalimab achieved an ORR of 32.3% and an mOS of 20.3 months [120]. A phase II study (NCT03951597) demonstrated for the first time that the combination of toripalimab with GEMOX and lenvatinib achieved an ORR of 80% in 30 iCCA patients, revealing that it could represent a promising treatment option [121]. Based on these findings, a confirmatory Phase III clinical trial (NCT05342194) has been initiated to validate the activity of the triple combination therapy comprising toripalimab, lenvatinib, and GEMOX chemotherapy. The ZSAB-TransGOLP study further demonstrated the powerful conversion potential of this triple regimen in locally advanced unresectable patients, achieving a 63% R0 resection rate and a median OS of 30.8 months [122]. This highlights the combination’s significant potential in achieving tumor downstaging and enabling conversion surgery. These advances underscore the broad research prospects for personalized precision therapy based on molecular subtyping.

### 5.5. Cancer Vaccines

Tumor-associated antigens (TAAs), characterized by their tumor-specific expression profiles, represent ideal targets for cancer vaccines due to their capacity to induce antigen-specific T-cell responses. Tumor cells frequently evade immune recognition through downregulation of major histocompatibility complex class I, and in intrahepatic cholangiocarcinoma, aberrant MHC-I expression has been associated with advanced disease progression, though the precise mechanisms remain poorly defined [70,123]. Multiple TAAs have been investigated as potential therapeutic targets in biliary tract cancers, including Wilms tumor 1 (WT1) and mucin 1 (MUC1). Both are reportedly expressed in about 80–85% of iCCA patients and correlate with unfavorable prognosis [124,125].

An open-label phase I clinical trial involving four iCCA patients demonstrated that combination therapy with WT1 peptide vaccine and gemcitabine yielded a mOS of 384 days, with evidence of immune activation in a subset of patients. The vaccine exhibited favorable tolerability without severe immune-related adverse events. However, the limited sample size and indeterminate clinical efficacy underscore the necessity for future studies to prioritize expanding cohort sizes, optimizing patient selection through WT1 expression profiling, and refining therapeutic protocols to enhance treatment outcomes [126].

Lepisto et al. conducted a phase I/II trial of a MUC1 peptide-pulsed autologous dendritic cell vaccine as adjuvant therapy in 12 patients with resected pancreatic and biliary tumors. Among the enrolled patients, two had cholangiocarcinoma. In these two patients, the disease-free survival was 16.7 months and 4.6 months, with OS of 23.4 months and 21.1 months. Notably, one iCCA patient remained recurrence-free during follow-up [127]. However, because this study included mixed tumor entities and only two cholangiocarcinoma cases, these findings should be interpreted as preliminary observations rather than iCCA-specific evidence of clinical efficacy. Nevertheless, the results suggest the feasibility and potential immunological relevance of dendritic cell-based vaccines in CCA, warranting further validation in larger, iCCA-focused cohorts.

Markus W. Löffler et al. reported a case of metastatic iCCA treated with a personalized multi-peptide vaccine carrying non-mutated epitopes, which elicited robust immune responses and achieved long-term tumor-free survival. Post-vaccination, functional T-cell responses against three of the seven peptides were observed [128]. More recently, the same group reported a long-term follow-up of this exceptional case. After repeated surgical resections and two successive personalized peptide vaccines, the patient remained tumor-free for more than 8 years. Importantly, long-term immune monitoring showed that vaccine-specific T-cell responses, predominantly CD4^+^ T-cell responses, persisted for years and were still detectable 5 years after the last vaccine administration. Spontaneous tumor neoantigen-specific CD4^+^ and CD8^+^ T-cell responses were also detected, suggesting that durable antitumor T-cell immunity may have contributed to the unusually favorable clinical course [129]. Although this remains a single-patient observation, it provides informative evidence for the durability of vaccine-induced T-cell responses in iCCA and supports further investigation of personalized peptide vaccination strategies. This represents the first documented use of adjuvant personalized peptide immunotherapy in cholangiocarcinoma, highlighting its potential as a promising novel therapeutic approach for iCCA. Further clinical validation is warranted to confirm its efficacy.

Koichi Shimizu et al. evaluated the efficacy of autologous tumor lysate-pulsed DC injections combined with CD3-activated T cell (CAT) transfer in postoperative iCCA patients. The CATs were generated ex vivo by stimulation with an anti-CD3 monoclonal antibody followed by IL-2 activation. The study enrolled 62 patients who underwent curative resection, with 36 receiving adjuvant DC vaccination plus CAT infusion (ATVAC group) and 26 serving as surgery-only controls. Compared to the controls, the ATVAC group demonstrated significantly improved PFS (18.3 vs. 7.7 months) and OS (31.9 vs. 17.4 months). Furthermore, early-stage (I/II) iCCA patients receiving ATVAC exhibited superior PFS and OS compared to stage IV patients, suggesting that this combination therapy enhances survival outcomes, particularly in early-stage iCCA [130].

### 5.6. Adoptive Cell Therapy

Adoptive cell therapy (ACT), a promising approach to inducing antitumor immune responses, involves the ex vivo expansion and engineering of T cells to confer tumor-specific recognition, followed by reinfusion of these amplified tumor-reactive lymphocytes into patients. Three primary ACT modalities are utilized in cancer treatment: tumor-infiltrating lymphocytes (TILs), T-cell receptor (TCR)-engineered T cells, and chimeric antigen receptor (CAR)-T-cell therapy [131]. In intrahepatic cholangiocarcinoma, ACT remains in early-stage exploration, with current evidence limited to small case series, single-patient reports, and single-arm pilot studies.

Ryota Higuchi et al. reported a case of a female iCCA patient with lymph node metastasis who achieved prolonged survival without recurrence after surgery combined with postoperative immunotherapy [132]. The patient initiated immunotherapy (CD3-activated T-cell infusions and tumor lysate/peptide-pulsed dendritic cell injections) two months post-surgery, remaining recurrence-free for 3.6 years with favorable clinical status. Eric Tran et al. successfully identified and expanded CD4^+^ T cells targeting tumor-specific mutations in a metastatic cholangiocarcinoma patient [133]. After two adoptive cell infusions, significant regression of hepatic and pulmonary lesions was observed, with disease control sustained for over 13 months. This study represents the first demonstration of CD4^+^ T-cell-mediated antitumor activity in cholangiocarcinoma, challenging the conventional CD8^+^ T-cell-centric paradigm and underscoring ACT’s clinical potential. However, the single-patient design limits generalizability, necessitating further validation in larger cohorts. A phase I trial (NCT01935843) evaluating HER2-targeted CAR-T cells in 11 advanced cholangiocarcinoma and pancreatic cancer patients demonstrated encouraging safety and feasibility of CAR-T-HER2 immunotherapy [134]. Kamonlapat Supimon et al. developed anti-MUC1-CAR4 T cells, which exhibited potent in vitro antitumor activity against MUC1-expressing cholangiocarcinoma cells [135]. As described above in the Section 5.5 [130], Shimizu et al. administered autologous tumor lysate-pulsed dendritic cells combined with adoptive transfer of ex vivo-activated T cells to resected ICC patients, achieving nearly doubled median overall survival (31.9 vs. 17.4 months) compared to surgery-alone controls. Additionally, an ongoing Thai clinical trial (NCT01868490) is investigating adoptive immunotherapy with modified autologous cytokine-induced killer (CIK) cells in patients with selected solid tumors, including cholangiocarcinoma. CIK cells are ex vivo expanded immune effector cells, with the CD3^+^CD56^+^ subset representing a major cytotoxic population. These cells exhibit MHC-unrestricted antitumor activity, and preclinical studies suggested that dendritic cell-exposed CIK cells may enhance antitumor cytotoxicity against cholangiocarcinoma [136]. This trial provides a relevant example of early clinical exploration of autologous CIK-cell transfer in cholangiocarcinoma.

A major limitation of ACT lies in its inherently personalized nature, requiring bespoke therapeutic agents tailored to individual patients. Nevertheless, ACT holds significant potential to advance precision medicine in ICC, offering novel therapeutic avenues for this aggressive malignancy.

## 6. Conclusions

ICCA remains a challenging cancer with limited effective treatment options. Despite advancements in immunotherapy, including immune checkpoint inhibitors and combination strategies, clinical responses remain suboptimal, reflecting the complexity of the iCCA immune microenvironment. The immunosuppressive features of iCCA, such as low immune cell infiltration, high levels of tumor-associated macrophages, myeloid-derived suppressor cells, and the upregulation of inhibitory receptors like PD-1/PD-L1, contribute to immune evasion and therapeutic resistance. Additionally, driver gene mutations such as *FGFR*2, *IDH*1/2, and *KRAS* play significant roles in modulating the immune landscape and influencing treatment response.

Emerging strategies such as combining ICIs with chemotherapy, targeted therapy, bispecific antibodies, and cancer vaccines are showing promise in preclinical and early clinical trials, providing new hope for patients. ICI plus chemotherapy, represented by TOPAZ-1 and KEYNOTE-966, currently has the strongest clinical support and has changed the first-line treatment landscape for advanced biliary tract cancer, including iCCA. In contrast, targeted therapy plus ICI, dual checkpoint blockade, bispecific antibodies, cancer vaccines, TILs, CAR-T, and TCR-T remain largely supported by early-phase, small-sample, single-arm, biomarker-selected, or non-iCCA-specific studies. Therefore, while these approaches show encouraging therapeutic potential, their clinical value should be further defined through iCCA-focused studies with careful assessment of survival benefit, toxicity, safety management, and patient selection. For vaccine- and cell-based therapies in particular, major challenges include the identification of iCCA-specific and immunogenic antigens, intratumoral heterogeneity that may lead to antigen loss or immune escape, and HLA restriction that limits the applicability of some TCR- or peptide-based strategies. In addition, insufficient T-cell trafficking into tumor tissues, dense desmoplastic stromal barriers, and potential on-target/off-tumor toxicity remain important obstacles to the safe and effective translation of vaccines, TILs, CAR-T, and TCR-T therapies in iCCA. Future iCCA-focused trials should incorporate molecular features, immune phenotypes, TLS status, stromal signatures, antigen-presentation capacity, and established biomarkers such as MSI-H/dMMR, TMB, and PD-L1 expression to identify patients most likely to benefit from specific immunotherapy combinations. A clearer hierarchy of evidence and biomarker-stratified trial design will be essential for translating these emerging strategies into clinically meaningful benefit in iCCA.

Future research should move beyond broad application of immunotherapy and toward more precise immune stratification in iCCA. Establishing an iCCA-specific immunophenotyping system that integrates lymphoid infiltration, myeloid dominance, TLS status, stromal exclusion, antigen-presentation capacity, and molecular alterations will be essential for guiding treatment selection. Biomarker-driven clinical trials should be designed to evaluate molecular subtype-specific immunotherapy combinations, particularly in *FGFR*2-, *IDH*1-, and *KRAS*-altered tumors. Advanced technologies, including single-cell sequencing, spatial transcriptomics, and multiplex imaging, may help define the spatial organization and functional states of immune cells within the iCCA tumor microenvironment. In parallel, myeloid-targeted therapies, stroma-remodeling strategies, and immune-priming approaches should be further explored to overcome immune exclusion and resistance to ICI-based therapy. Future trials should also incorporate dynamic immune monitoring and post-resistance re-biopsy analyses to better understand treatment adaptation, acquired resistance, and rational subsequent combination strategies.

## Figures and Tables

**Table 1:** Published Clinical trials of bispecific antibodies and dual checkpoint blockade in intrahepatic cholangiocarcinoma.

NCT Number (Study Name)	Regimen	Line of Therapy	Study Design	Tumor Type/iCCA Proportion	Key Efficacy Data	Grade ≥ 3 Toxicity	Major Limitations
NCT02923934 (CA209-538)	Nivolumab + Ipilimumab	Mostly later-line	Phase II, nonrandomized rare-cancer trial; BTC subgroup analysis	Advanced BTC; iCCA 16/39 (41%)	ICCA cohort: ORR 31%, DCR 43.75%;	Grade 3/4 immune-related AEs: 15%	Small BTC subgroup; nonrandomized design; not iCCA-specific
NCT04066491	Bintrafusp Alfa + GemCis vs. GemCis	First-line	Randomized phase II/III	Advanced BTC; iCCA 137/309 (44.3%)	No survival benefit; mPFS 5.5 vs. 5.6 months; mOS 11.5 vs. 11.5 months	Grade ≥ 3 AEs: 68.5% vs. 74.5%; most common grade ≥ 3 TRAEs included anemia 26.0% vs. 22.8%; bleeding AEs were more frequent with Bintrafusp Alfa (28.8% vs. 7.4%), with grade ≥ 3 bleeding events in 7.5%.	Negative trial; failed to improve OS/PFS despite biological rationale; iCCA-specific outcomes not separately reported
NCT05023109 (ZSAB-TOP)	Tislelizumab + Ociperlimab + GemCis	First-line	Multicenter, single-arm phase II	Advanced BTC; iCCA 75.6% (34/45)	cORR 48.8% (20/41), mPFS 7.7 months, mOS not reached	Grade ≥ 3 TRAEs: 60.0% (27/45)	Single-arm design; mixed BTC population

Table 1 summarizes published clinical trials or studies with available results evaluating bispecific antibodies and dual checkpoint blockade in patients with intrahepatic cholangiocarcinoma. Abbreviations: NCT, National Clinical Trial; GemCis, gemcitabine and cisplatin; BTC, biliary tract cancer; iCCA, intrahepatic cholangiocarcinoma; ORR, objective response rate; DCR, disease control rate; cORR, confirmed objective response rate; PFS, progression-free survival; OS, overall survival; mPFS, median progression-free survival; mOS, median overall survival; AE, adverse event; TRAE, treatment-related adverse event.

**Table 2:** Ongoing or registered clinical trials of bispecific antibodies and dual checkpoint blockade in intrahepatic cholangiocarcinoma.

NCT Number	Study Design	Conditions	Therapeutic Strategy	Target	Line of Therapy	Primary Endpoint or Key Endpoint
NCT06467357	Phase III, randomized, open-label	HER2-expressing advanced BTC	Trastuzumab Deruxtecan and Rilvegostomig	HER2, PD-1/TIGIT	First-line	OS
NCT05023109	Phase II, multicenter, single-arm	Advanced BTC	GemCis + Tislelizumab + Ociperlimab	PD-1, TIGIT	First-line	ORR
NCT07129018	Phase II, prospective, open-label	HER2-expressing locally advanced or metastatic BTC	Trastuzumab-rezetecan + Pertuzumab + Iparomlimab and Tuvonralimab	HER2, PD-1/CTLA-4	First-line	ORR
NCT05653180	Phase I/II	Advanced BTC	IBI310 + Sintilimab	CTLA-4, PD-1	Advanced/previously treated	Safety (DLTs, grade ≥ 3 AEs) and ORR
NCT04238637	Phase II, randomized, prospective, open-label	Advanced iCCA	Durvalumab + Tremelimumab + Y-90 SIRT	PD-L1, CTLA-4	Locoregional/advanced disease	ORR
NCT03937830	Phase I/II	Advanced BTC or HCC	Bevacizumab + Durvalumab + Tremelimumab (+TACE)	VEGF, PD-L1, CTLA-4	Advanced/previously treated	Safety (DLTs, grade ≥ 3 AEs) and preliminary efficacy
NCT03704480	Phase I	Advanced solid tumors including BTC	Durvalumab + Tremelimumab (+Paclitaxel)	PD-L1, CTLA-4	Advanced/previously treated	Safety and tolerability (DLTs, grade ≥ 3 AEs)
NCT05297903	Phase II, single-arm	Advanced BTC including iCCA	XmAb20717	PD-1, CTLA-4	Later-line after gemcitabine-based chemotherapy	ORR

Table 2 provides an overview of ongoing or registered clinical trials testing bispecific antibodies and dual checkpoint blockade in intrahepatic cholangiocarcinoma, as listed on ClinicalTrials.gov. Abbreviations: PD-1, programmed cell death protein 1; PD-L1, programmed death-ligand 1; CTLA-4, cytotoxic T-lymphocyte-associated protein 4; TIGIT, T-cell immunoreceptor with Ig and ITIM domains; HER2, human epidermal growth factor receptor 2; VEGF, vascular endothelial growth factor; Y-90 SIRT, yttrium-90 selective internal radiation therapy; TACE, transarterial chemoembolization; DLTs, dose-limiting toxicities; HCC, hepatocellular carcinoma.

**Table 3:** Published clinical trials of immune checkpoint inhibitors combined with chemotherapy in intrahepatic cholangiocarcinoma.

NCT Number (Study Name)	Regimen	Line of Therapy	Study Design	Tumor Type/ iCCA Proportion	Key Efficacy Data	Grade ≥ 3 Toxicity	Major Limitations
NCT03046862	Durvalumab + GemCis with or without Tremelimumab	First-line	Open-label, single-center phase II	Advanced BTC; iCCA proportion NR	ORR 72% with GemCis + durvalumab and 70% with GemCis + durvalumab + tremelimumab; mPFS 11.8 and 12.3 months; mOS 20.2 and 18.7 months, respectively	Decreased neutrophil count 53%, anemia 40%, and decreased platelet count 19%	Single-center phase II study; limited sample size; not iCCA-specific
NCT03875235 (TOPAZ-1)	Durvalumab + GemCis vs. Placebo + GemCis	First-line	Global phase III, randomized, double-blind, placebo-controlled	Advanced BTC; iCCA 383/685 (56%)	ORR 26.7% vs. 18.7%; mPFS 7.2 vs. 5.7 months; mOS 12.8 vs. 11.5 months	Grade 3/4 TRAEs: 62.7% vs. 64.9%	ICCA subgroup supportive but not iCCA-only
NCT04003636 (KEYNOTE-966)	Pembrolizumab + GemCis vs. Placebo + GemCis	First-line	Global phase III, randomized, double-blind, placebo-controlled	Advanced BTC; iCCA 633/1069 (59.2%)	ORR 29% vs. 29%; mPFS 6.5 vs. 5.6 months; mOS 12.7 vs. 10.9 months	Grade 3/4 AEs: 79% vs. 75%	ORR not improved; iCCA subgroup supportive but not iCCA-only
NCT03111732	Pembrolizumab + CAPOX	Mixed line; mostly previously treated or refused first-line therapy	Single-arm phase II	Advanced BTC; iCCA 8/11 (73%)	PR 27.3%; SD 54%; DCR 81.8%; mPFS 4.1 mo; mOS 9.9 months	Decreased lymphocyte count 64%, anemia 36%, decreased platelet count 27%, and hyponatremia 27%	Small study; single-arm design; mixed treatment lines

Table 3 summarizes published clinical trials with available results evaluating immune checkpoint inhibitors combined with chemotherapy in intrahepatic cholangiocarcinoma. Abbreviations: NR, not reported; CAPOX, capecitabine and oxaliplatin; PR, partial response; SD, stable disease; mo, months.

**Table 4:** Ongoing or registered clinical trials of immune checkpoint inhibitors combined with chemotherapy in intrahepatic cholangiocarcinoma.

NCT Number	Study Design	Conditions	Therapeutic Strategy	Target	Line of Therapy	Primary Endpoint or Key Endpoint
NCT06988592	Prospective, non-interventional, observational study	Advanced Biliary Tract Cancer	PD-1/PD-L1 inhibitor + GemCis	PD-1/PD-L1	First-line	OS, PFS
NCT06903273	Phase II, single-arm, prospective	Cholangiocarcinoma, Biliary Tract Cancer	Tislelizumab + GemCis + S-1	PD-1	First-line	ORR
NCT06893380	Phase Ib/II, multicenter, open-label	Locally Advanced Biliary Tract Cancers, Metastatic Biliary Tract Cancers	GemCis + Nab-paclitaxel + Tislelizumab	PD-1	First-line	PFS
NCT06490107	Phase II, single-center, single-arm, prospective	Biliary Tract Cancer	Durvalumab + S-1	PD-L1	Protocol-defined	ORR
NCT05924880	Phase IIIb, open-label, single-arm, multicenter	Biliary Tract Cancers	Durvalumab + Gemcitabine + Oxalitin/S-1/Cisplatin	PD-L1	Protocol-defined	OS
NCT05822453	Phase II, single-arm	Biliary Tract Carcinoma	Gemcitabine + S-1 + Tislelizumab	PD-1	First-line	ORR
NCT03486678	Single-arm, open-label, exploratory phase II	Biliary Tract Cancer, Cholangiocarcinoma	SHR-1210 + GEMOX	PD-1	First-line or protocol-defined	ORR
NCT03478488	Phase III, randomized, open-label, multicenter	Biliary Tract Neoplasms	KN035 + GEMOX	PD-L1	First-line or protocol-defined	OS

Table 4 provides an overview of ongoing or registered clinical trials testing immune checkpoint inhibitors combined with chemotherapy in intrahepatic cholangiocarcinoma, as listed on ClinicalTrials.gov. Abbreviations: S-1, oral fluoropyrimidine S-1; GEMOX, gemcitabine plus oxaliplatin; SHR-1210, camrelizumab; KN035, envafolimab.

**Table 5:** Published clinical trials of immune checkpoint inhibitors plus targeted therapy with or without chemotherapy in intrahepatic cholangiocarcinoma.

NCT Number (Study Name)	Regimen	Line of Therapy	Study Design	Tumor Type/iCCA Proportion	Key Efficacy Data	Grade ≥ 3 Toxicity	Major Limitations
NCT04677504 (IMbrave151)	Atezolizumab + bevacizumab + GemCis vs. atezolizumab + placebo + GemCis	First-line	Global phase II, randomized, double-blind	Advanced BTC; iCCA 54%	iCCA subgroup: mPFS 8.3 vs. 8.3 months; mOS 16.7 vs. 17.2 months	Grade 3/4 AEs: 73% vs. 74%	No OS or ORR benefit
NCT04361331 (zs-ICC-2020)	Lenvatinib + toripalimab	First-line	Two-cohort, single-center, open-label phase II	Advanced iCCA	ORR 32.3%; mPFS 8.9 mo; mOS 20.3 months	Grade 3/4 AEs: 35.5%	Small sample; no GemCis control
NCT03951597	Toripalimab + lenvatinib + GEMOX	First-line	Single-arm phase II	Advanced iCCA	ORR 80.0%; mPFS 10.2 mo; mOS 22.5 months	Grade ≥ 3 AEs: 56.7%	Single-arm; selection bias; needs phase III validation
NCT05156788 (ZSAB-TransGOLP)	Tislelizumab + lenvatinib + GEMOX	Conversion therapy	Multicenter prospective phase II	Unresectable BTC; iCCA 87.8%	R0 resection rate 63%; mOS 30.8 months	Grade 3–4 TRAEs 49%	Conversion setting; not comparable to metastatic first-line
NCT04056910	Ivosidenib + nivolumab	Later-line	Phase II	*IDH*1-mutant solid tumors including BTC; cholangiocarcinoma 4/15 (26.7%); iCCA proportion NR	Overall: 3/15 (20%); mPFS 1.94 months; ORR 6.7%	Grade ≥ 3 TRAEs: 27%	Small biomarker-selected cohort
NCT05921760	Ivosidenib + nivolumab + ipilimumab	Later-line	Phase II	Nonresectable or metastatic *IDH*1-mutant CCA; iCCA 7/7 (100%)	ORR 0%; SD 3/7; PD 4/7; expansion phase not conducted	All patients experienced Grade ≥ 3 TEAEs and treatment-related Grade ≥ 3 TEAEs	Small exploratory study

Table 5 summarizes published clinical trials or studies with available results evaluating ICIs plus targeted therapy with or without chemotherapy in intrahepatic cholangiocarcinoma. Abbreviations: R0, microscopically margin-negative resection; *IDH*1, isocitrate dehydrogenase 1; CCA, cholangiocarcinoma; PD, progressive disease; TEAEs, treatment-emergent adverse events.

**Table 6:** Ongoing or registered clinical trials of immune checkpoint inhibitors plus targeted therapy with or without chemotherapy in intrahepatic cholangiocarcinoma.

NCT Number	Study Design	Conditions	Therapeutic Strategy	Target	Line of Therapy	Primary Endpoint or Key Endpoint
NCT05342194	Phase III, randomized, double-blind	Advanced iCCA	Toripalimab + Lenvatinib + GEMOX	PD-1 + VEGFR	First-line	PFS
NCT06648057	Prospective observational/real-world biomarker study	Biliary Tract Cancer, Cholangiocarcinoma	Lenvatinib + Pembrolizumab	PD-1, VEGFR/FGFR	Protocol-defined	ORR, PFS, OS
NCT07111546	Phase II, open-label, multicenter study	Advanced Solid Tumor	LBL-024 + Bevacizumab	PD-L1/4-1BB, VEGF	Protocol-defined	ORR
NCT03768531	Phase II, randomized perioperative study	Resectable Biliary Tract Cancer	Nivolumab + Cabiralizumab	PD-1, CSF-1R	Neoadjuvant and adjuvant/perioperative	DFS, OS
NCT03092895	Phase II, open-label, nonrandomized study	Advanced Primary Liver Cancer, Advanced Biliary Tract Carcinoma	SHR-1210 + Apatinib	PD-1, VEGFR	Protocol-defined	ORR, PFS
NCT07062328	Phase II, open-label study	Biliary Tract Carcinoma	Recaticimab + Adebrelimab + Chemotherapy	PD-L1, PCSK9	First-line for unresectable/metastatic BTC	ORR
NCT06963060	Phase II	Gallbladder Cancer, Cholangiocarcinoma	Gemcitabine + Nab-paclitaxel + Lenvatinib + Tislizumab	PD-1, VEGFR/FGFR	First-line/advanced unresectable setting	ORR
NCT06320301	Phase II, open-label, single-arm study	Biliary Tract Cancer	Adebulimab + GEMOX + TKI	PD-L1, VEGFR	First-line	ORR
NCT06037655	Phase II	Biliary Tract Neoplasms	GemCis+ Adebrelimab + Mecapegfilgrastim	PD-L1, CSF-3R	Neoadjuvant	ORR
NCT05742750	Phase I/II	Locally Advanced Biliary Tract Cancer, Metastatic Biliary Tract Cancer	Camrelizumab + Apatinib + GemCis	PD-1, VEGFR	First-line	DLT, MTD, ORR
NCT05410197	Phase II, prospective, open-label, single-arm study	Advanced Biliary Tract Cancer	Envofolimab + Lenvatinib + GemCis	PD-L1, VEGFR/FGFR	First-line	ORR
NCT05327582	Phase I/II, open-label, single-arm study	Pancreatic Cancer, Biliary Tract Cancer	Durvalumab + Lenvatinib + Paclitaxel albumin	PD-L1, VEGFR/FGFR	Protocol-defined	ORR, DLT
NCT05254847	Phase II, prospective, open-label, single-center study	Biliary Tract Cancer	Capecitabine + Lenvatinib + Tislelizumab	PD-1, VEGFR/FGFR	Protocol-defined	RFS, DFS
NCT04217954	Phase II	Advanced Biliary Tract Cancer	Oxaliplatin + 5-FU + Bevacizumab + Toripalimab	PD-1, VEGF	Protocol-defined	ORR

Table 6 provides an overview of ongoing or registered clinical trials testing ICIs plus targeted therapy with or without chemotherapy in intrahepatic cholangiocarcinoma, as listed on ClinicalTrials.gov. Abbreviations: 5-FU, 5-fluorouracil; CSF-1R, colony-stimulating factor 1 receptor; CSF-3R, colony-stimulating factor 3 receptor; DFS, disease-free survival; DLT, dose-limiting toxicity; FGFR, fibroblast growth factor receptor; MTD, maximum tolerated dose; PCSK9, proprotein convertase subtilisin/kexin type 9; RFS, recurrence-free survival; TKI, tyrosine kinase inhibitor; VEGFR, vascular endothelial growth factor receptor. 4-1BB, tumor necrosis factor receptor superfamily member 9.

## Data Availability

No new data were created or analyzed in this study. Data sharing is not applicable to this article.

## References

[1] Sigel C.S., Drill E., Zhou Y., Basturk O., Askan G., Pak L.M., Vakiani E., Wang T., Boerner T., Do R.K. (2018). Intrahepatic Cholangiocarcinomas Have Histologically and Immunophenotypically Distinct Small and Large Duct Patterns. Am. J. Surg. Pathol..

[2] De Santis A., Zhu L., Tao J., Reißfelder C., Schölch S. (2025). Molecular subtypes of intrahepatic cholangiocarcinoma. Trends Mol. Med..

[3] Nagtegaal I.D., Odze R.D., Klimstra D., Paradis V., Rugge M., Schirmacher P., Washington K.M., Carneiro F., Cree I.A., the WHO Classification of Tumours Editorial Board (2020). The 2019 WHO classification of tumours of the digestive system. Histopathology.

[4] Chung T., Rhee H., Nahm J.H., Jeon Y., Yoo J.E., Kim Y.-J., Han D.H., Park Y.N. (2020). Clinicopathological characteristics of intrahepatic cholangiocarcinoma according to gross morphologic type: Cholangiolocellular differentiation traits and inflammation- and proliferation-phenotypes. HPB.

[5] Hou W. (2025). Role of TGFβ-activated cancer-associated fibroblasts in the resistance to checkpoint blockade immunotherapy. Front. Oncol..

[6] Fabris L., Perugorria M.J., Mertens J., Björkström N.K., Cramer T., Lleo A., Solinas A., Sänger H., Lukacs-Kornek V., Moncsek A. (2019). The tumour microenvironment and immune milieu of cholangiocarcinoma. Liver Int..

[7] Kendre G., Murugesan K., Brummer T., Segatto O., Saborowski A., Vogel A. (2022). Charting co-mutation patterns associated with actionable drivers in intrahepatic cholangiocarcinoma. J. Hepatol..

[8] Eluri M., De Armas A.D., Ross J.S., Sharaf R., Pavlick D.C., Chatterjee D., Javle M.M. (2023). Clinical and mutational profiles of microsatellite instability (MSI-H) in intrahepatic cholangiocarcinoma (iCCA). J. Clin. Oncol..

[9] Tan S., Feng M., Zhou N., Zhang S., Yi C., Gou H. (2025). DNA damage response and repair gene mutations predict clinical outcomes in biliary tract cancer. Cancer.

[10] Zhang L., Zheng H., Jiang S.-T., Liu Y.-G., Zhang T., Zhang J.-W., Lu X., Zhao H.-T., Sang X.-T., Xu Y.-Y. (2024). Worldwide research trends on tumor burden and immunotherapy: A bibliometric analysis. Int. J. Surg..

[11] Bertuccio P., Malvezzi M., Carioli G., Hashim D., Boffetta P., El-Serag H.B., La Vecchia C., Negri E. (2019). Global trends in mortality from intrahepatic and extrahepatic cholangiocarcinoma. J. Hepatol..

[12] Cillo U., Fondevila C., Donadon M., Gringeri E., Mocchegiani F., Schlitt H.J., Ijzermans J.N.M., Vivarelli M., Zieniewicz K., Damink S.W.M.O. (2019). Surgery for cholangiocarcinoma. Liver Int..

[13] Ratti F., Maina C., Aldrighetti L.A.M. (2024). ASO Author Reflections: Minimally Invasive Approach and Oncologic Benefit in Intrahepatic Cholangiocarcinoma (iCCA) With Risk of Very Early Recurrence: Is it Time to Consider Technique as a Key Element of Onco-Surgical Strategy?. Ann. Surg. Oncol..

[14] Song Y., Huang S.-W., Shu B., Zhou Y.-X., Dai W.-D., Sun B.-Y. (2025). Gemcitabine-cisplatin chemotherapy plus anti-PD-L1 therapy reinvigorates antitumor immune response by reprogramming the intrahepatic cholangiocarcinoma microenvironment. Front. Immunol..

[15] Szeto G.L., Finley S.D. (2019). Integrative Approaches to Cancer Immunotherapy. Trends Cancer.

[16] Job S., Rapoud D., Dos Santos A., Gonzalez P., Desterke C., Pascal G., Elarouci N., Ayadi M., Adam R., Azoulay D. (2019). Identification of Four Immune Subtypes Characterized by Distinct Composition and Functions of Tumor Microenvironment in Intrahepatic Cholangiocarcinoma. Hepatology.

[17] Alvisi G., Termanini A., Soldani C., Portale F., Carriero R., Pilipow K., Costa G., Polidoro M., Franceschini B., Malenica I. (2022). Multimodal single-cell profiling of intrahepatic cholangiocarcinoma defines hyperactivated Tregs as a potential therapeutic target. J. Hepatol..

[18] Zimmer C.L., Filipovic I., Cornillet M., O’rOurke C.J., Berglin L., Jansson H., Sun D., Strauss O., Hertwig L., Johansson H. (2021). Mucosal-associated invariant T-cell tumor infiltration predicts long-term survival in cholangiocarcinoma. Hepatology.

[19] Milardi G., Franceschini B., Camisaschi C., Puccio S., Costa G., Soldani C., Uva P., Cangelosi D., Carriero R., Lambroia L. (2025). Immunosuppressive contribution of tumour-infiltrating B cells in human intrahepatic cholangiocarcinoma and their role in chemoimmunotherapy outcome. Gut.

[20] Doi N., Ino Y., Fuse M., Esaki M., Shimada K., Hiraoka N. (2023). Correlation of Vein-Rich Tumor Microenvironment of Intrahepatic Cholangiocarcinoma With Tertiary Lymphoid Structures and Patient Outcome. Mod. Pathol..

[21] Ye Y.-H., Xin H.-Y., Li N., Luo C.-B., Chen L., Pan J.-Y., Xu Y., Weng F., Tu C.-Y., Ji Y.-Y. (2025). The intratumoral balance of IgG4^+^ plasma cells and CD8^+^ T cells is associated with prognosis of intrahepatic cholangiocarcinoma after curative resection. Dig. Liver Dis..

[22] Polidoro M.A., Mikulak J., Cazzetta V., Lleo A., Mavilio D., Torzilli G., Donadon M. (2020). Tumor microenvironment in primary liver tumors: A challenging role of natural killer cells. World J. Gastroenterol..

[23] Oliviero B., Varchetta S., Mele D., Pessino G., Maiello R., Falleni M., Tosi D., Donadon M., Soldani C., Franceschini B. (2022). MICA/B-targeted antibody promotes NK cell–driven tumor immunity in patients with intrahepatic cholangiocarcinoma. OncoImmunology.

[24] Fukuda Y., Asaoka T., Eguchi H., Yokota Y., Kubo M., Kinoshita M., Urakawa S., Iwagami Y., Tomimaru Y., Akita H. (2019). Endogenous CXCL9 affects prognosis by regulating tumor-infiltrating natural killer cells in intrahepatic cholangiocarcinoma. Cancer Sci..

[25] Meng X.-L., Lu J.-C., Zhang Y.-X., Pu P., Guo X.-J., Zhu T., Hu Z.-Q., Yu L., Sun Q.-M., Gao Q. (2026). Norepinephrine promotes tumour cell aggressiveness and NK cell ferroptosis via ADRB2 in intrahepatic cholangiocarcinoma with perineural invasion. Gut.

[26] Zhang Q.-W., Zhu M.-X., Liu W.-F., Rui W.-W., Chen Y., Ding X.-Y., Jiang Y.-S., Wu Z.-Y., Liu B.-B. (2024). Identification of clinically relevant subsets CD39^+^PD-1^+^CD8^+^ T cells and CD39^+^ regulatory T cells in intrahepatic cholangiocarcinoma using single-cell CyTOF. Transl. Oncol..

[27] Toledo B., Chen L.Z., Paniagua-Sancho M., Marchal J.A., Perán M., Giovannetti E. (2024). Deciphering the performance of macrophages in tumour microenvironment: A call for precision immunotherapy. J. Hematol. Oncol..

[28] Bernatz S., Schulze F., Bein J., Bankov K., Mahmoudi S., Grünewald L.D., Koch V., Stehle A., Schnitzbauer A.A., Walter D. (2024). Small duct and large duct type intrahepatic cholangiocarcinoma reveal distinct patterns of immune signatures. J. Cancer Res. Clin. Oncol..

[29] Diggs L.P., Ruf B., Ma C., Heinrich B., Cui L., Zhang Q., McVey J.C., Wabitsch S., Heinrich S., Rosato U. (2020). CD40-mediated immune cell activation enhances response to anti-PD-1 in murine intrahepatic cholangiocarcinoma. J. Hepatol..

[30] Chen M., Zhao S., Xie X., Wang J., Su M., Zhang L., Cui R., Zhao D. (2025). Role of Tunneling Nanotubes in Arachidonic Acid Transfer and Macrophage Function Reprogramming in Intrahepatic Cholangiocarcinoma. Adv. Sci..

[31] Dong Z.-R., Zhang M.-Y., Qu L.-X., Zou J., Yang Y.-H., Ma Y.-L., Yang C.-C., Cao X.-L., Wang L.-Y., Zhang X.-L. (2024). Spatial resolved transcriptomics reveals distinct cross-talk between cancer cells and tumor-associated macrophages in intrahepatic cholangiocarcinoma. Biomark. Res..

[32] Agirre-Lizaso A., Huici-Izagirre M., O’ROurke C.J., Zhuravleva E., Carpino G., Overi D., Urretabizkaia-Garmendia J., Labiano I., Korosec A., Val B. (2026). MARCO promotes cholangiocarcinogenesis by inducing immunosuppression and its targeting reduces tumor growth. Signal Transduct. Target. Ther..

[33] Jarman E.J., Horcas-Lopez M., Waddell S.H., MacMaster S., Gournopanos K., Soong D.Y.H., Musialik K.I., Tsokkou P., Ng M., Cambridge W.A. (2022). DKK1 drives immune suppressive phenotypes in intrahepatic cholangiocarcinoma and can be targeted with anti-DKK1 therapeutic DKN-01. Liver Int..

[34] Tomlinson J.L., Valle J.W., Ilyas S.I. (2023). Immunobiology of cholangiocarcinoma. J. Hepatol..

[35] Loeuillard E., Yang J., Buckarma E., Wang J., Liu Y., Conboy C., Pavelko K.D., Li Y., O’brien D., Wang C. (2020). Targeting tumor-associated macrophages and granulocytic myeloid-derived suppressor cells augments PD-1 blockade in cholangiocarcinoma. J. Clin. Investig..

[36] Sun B.-Y., Wang Z.-T., Chen K.-Z., Song Y., Wu J.-F., Zhang D., Sun G.-Q., Zhou J., Fan J., Hu B. (2024). Mobilization and activation of tumor-infiltrating dendritic cells inhibits lymph node metastasis in intrahepatic cholangiocarcinoma. Cell Death Discov..

[37] Vobořil M., Xuan S., Hogquist K.A. (2025). Thymic Dendritic Cells Revisited. Immunol. Rev..

[38] Pei D.-N., Song Y., Zhou Y.-X., Shu B., Huang S.-W., Li F.-Z., Dai W.-D., Sun B.-Y. (2025). Enhancing cDC1-mediated anti-tumor immunity limits tumor progression and potentiates anti-PD-1 therapy in intrahepatic cholangiocarcinoma. Front. Immunol..

[39] Li K., Shi H., Zhang B., Ou X., Ma Q., Chen Y., Shu P., Li D., Wang Y. (2021). Myeloid-derived suppressor cells as immunosuppressive regulators and therapeutic targets in cancer. Signal Transduct. Target. Ther..

[40] Bozkus C.C., Elzey B.D., Crist S.A., Ellies L.G., Ratliff T.L. (2015). Expression of Cationic Amino Acid Transporter 2 Is Required for Myeloid-Derived Suppressor Cell–Mediated Control of T Cell Immunity. J. Immunol..

[41] Noman M.Z., Desantis G., Janji B., Hasmim M., Karray S., Dessen P., Bronte V., Chouaib S. (2014). PD-L1 is a novel direct target of HIF-1α, and its blockade under hypoxia enhanced MDSC-mediated T cell activation. J. Exp. Med..

[42] Onishi Y., Fehervari Z., Yamaguchi T., Sakaguchi S. (2008). Foxp3^+^ natural regulatory T cells preferentially form aggregates on dendritic cells in vitro and actively inhibit their maturation. Proc. Natl. Acad. Sci. USA.

[43] Ricci A.D., Rizzo A., Schirizzi A., D’alessandro R., Frega G., Brandi G., Shahini E., Cozzolongo R., Lotesoriere C., Giannelli G. (2024). Tumor Immune Microenvironment in Intrahepatic Cholangiocarcinoma: Regulatory Mechanisms, Functions, and Therapeutic Implications. Cancers.

[44] Bergmann C., Wild C.A., Narwan M., Lotfi R., Lang S., Brandau S. (2011). Human tumor-induced and naturally occurring Treg cells differentially affect NK cells activated by either IL-2 or target cells. Eur. J. Immunol..

[45] Ghiringhelli F., Menard C., Terme M., Flament C., Taieb J., Chaput N., Puig P.E., Novault S., Escudier B., Vivier E. (2005). CD4+CD25+ regulatory T cells inhibit natural killer cell functions in a transforming growth factor-beta-dependent manner. J. Exp. Med..

[46] Wculek S.K., Cueto F.J., Mujal A.M., Melero I., Krummel M.F., Sancho D. (2020). Dendritic cells in cancer immunology and immunotherapy. Nat. Rev. Immunol..

[47] Ding G.-Y., Ma J.-Q., Yun J.-P., Chen X., Ling Y., Zhang S., Shi J.-Y., Chang Y.-Q., Ji Y., Wang X.-Y. (2022). Distribution and density of tertiary lymphoid structures predict clinical outcome in intrahepatic cholangiocarcinoma. J. Hepatol..

[48] Lin J., Dai Y., Sang C., Song G., Xiang B., Zhang M., Dong L., Xia X., Ma J., Shen X. (2022). Multimodule characterization of immune subgroups in intrahepatic cholangiocarcinoma reveals distinct therapeutic vulnerabilities. J. Immunother. Cancer.

[49] Cammarota A., Balsano R., Pressiani T., Bozzarelli S., Rimassa L., Lleo A. (2025). The Immune–Genomics of Cholangiocarcinoma: A Biological Footprint to Develop Novel Immunotherapies. Cancers.

[50] Moeini A., Sia D., Bardeesy N., Mazzaferro V., Llovet J.M. (2016). Molecular Pathogenesis and Targeted Therapies for Intrahepatic Cholangiocarcinoma. Clin. Cancer Res..

[51] Lin Y., Peng L., Dong L., Liu D., Ma J., Lin J., Chen X., Lin P., Song G., Zhang M. (2022). Geospatial Immune Heterogeneity Reflects the Diverse Tumor–Immune Interactions in Intrahepatic Cholangiocarcinoma. Cancer Discov..

[52] Baretti M., Shekhar S., Sahai V., Shu D., Howe K., Gunchick V., Assarzadegan N., Kartalia E., Zhu Q., Hallab E. (2025). Deep immune profiling of intrahepatic cholangiocarcinoma with CODEX multiplexed imaging. Hepatol. Commun..

[53] Liu S., Weng J., Cao M., Zhou Q., Xu M., Xu W., Hu Z., Xu M., Dong Q., Sheng X. (2024). *FGFR2* fusion/rearrangement is associated with favorable prognosis and immunoactivation in patients with intrahepatic cholangiocarcinoma. Oncologist.

[54] Saborowski A., Lehmann U., Vogel A. (2020). FGFR inhibitors in cholangiocarcinoma: What’s now and what’s next?. Ther. Adv. Med. Oncol..

[55] Notarangelo G., Spinelli J.B., Perez E.M., Baker G.J., Kurmi K., Elia I., Stopka S.A., Baquer G., Lin J.-R., Golby A.J. (2022). Oncometabolite d-2HG alters T cell metabolism to impair CD8^+^ T cell function. Science.

[56] Xu W., Yang H., Liu Y., Yang Y., Wang P., Kim S.-H., Ito S., Yang C., Wang P., Xiao M.-T. (2011). Oncometabolite 2-Hydroxyglutarate Is a Competitive Inhibitor of α-Ketoglutarate-Dependent Dioxygenases. Cancer Cell.

[57] Wu M., Shi L., Merritt J., Zhu A.X., Bardeesy N. (2022). Biology of IDH mutant cholangiocarcinoma. Hepatology.

[58] Wu M.-J., Shi L., Dubrot J., Merritt J., Vijay V., Wei T.-Y., Kessler E., Olander K.E., Adil R., Pankaj A. (2022). Mutant IDH Inhibits IFNγ–TET2 Signaling to Promote Immunoevasion and Tumor Maintenance in Cholangiocarcinoma. Cancer Discov..

[59] Zabransky D.J., Kartalia E., Lee J.W., Leatherman J.M., Charmsaz S., Young S.E., Chhabra Y., Franch-Expósito S., Kang M., Maru S. (2024). Tumor-derived CCL2 drives tumor growth and immunosuppression in IDH1-mutant cholangiocarcinoma. Hepatology.

[60] Zhang M., Huang Y., Pan J., Sang C., Lin Y., Dong L., Shen X., Wu Y., Song G., Ji S. (2023). An Inflammatory Checkpoint Generated by *IL1RN* Splicing Offers Therapeutic Opportunity for *KRAS*-Mutant Intrahepatic Cholangiocarcinoma. Cancer Discov..

[61] Lin J., Sang C., Xiang B., Shen X., Zhao J., Xu S., Pan J., Lin Y., Dong L., Chu Q. (2026). Arachidonic Acid Metabolism in PMN-MDSCs Suppresses Antitumor Capacity of T cells in KRAS-Mutant Cholangiocarcinoma. Cancer Discov..

[62] Deng M., Li S.-H., Fu X., Yan X.-P., Chen J., Qiu Y.-D., Guo R.-P. (2021). Relationship between PD-L1 expression, CD8+ T-cell infiltration and prognosis in intrahepatic cholangiocarcinoma patients. Cancer Cell Int..

[63] Xian F., Ren D., Bie J., Xu G. (2023). Prognostic value of programmed cell death ligand 1 expression in patients with intrahepatic cholangiocarcinoma: A meta-analysis. Front. Immunol..

[64] Bashyam H. (2007). CTLA-4: From conflict to clinic. J. Exp. Med..

[65] Rowshanravan B., Halliday N., Sansom D.M. (2018). CTLA-4: A moving target in immunotherapy. Blood.

[66] Guo X.-J., Lu J.-C., Zeng H.-Y., Zhou R., Sun Q.-M., Yang G.-H., Pei Y.-Z., Meng X.-L., Shen Y.-H., Zhang P.-F. (2021). CTLA-4 Synergizes With PD1/PD-L1 in the Inhibitory Tumor Microenvironment of Intrahepatic Cholangiocarcinoma. Front. Immunol..

[67] Heij L., Bednarsch J., Tan X., Rosin M., Appinger S., Reichel K., Pecina D., Doukas M., van Dam R.M., Vallejo J.G. (2023). Expression of Checkpoint Molecules in the Tumor Microenvironment of Intrahepatic Cholangiocarcinoma: Implications for Immune Checkpoint Blockade Therapy. Cells.

[68] Cai L., Li Y., Tan J., Xu L., Li Y. (2023). Targeting LAG-3, TIM-3, and TIGIT for cancer immunotherapy. J. Hematol. Oncol..

[69] Goeppert B., Frauenschuh L., Zucknick M., Roessler S., Mehrabi A., Hafezi M., Stenzinger A., Warth A., Pathil A., Renner M. (2015). Major histocompatibility complex class I expression impacts on patient survival and type and density of immune cells in biliary tract cancer. Br. J. Cancer.

[70] Sabbatino F., Villani V., Yearley J.H., Deshpande V., Cai L., Konstantinidis I.T., Moon C., Nota S., Wang Y., Al-Sukaini A. (2016). PD-L1 and HLA Class I Antigen Expression and Clinical Course of the Disease in Intrahepatic Cholangiocarcinoma. Clin. Cancer Res..

[71] Yoshihama S., Vijayan S., Sidiq T., Kobayashi K.S. (2017). NLRC5/CITA: A Key Player in Cancer Immune Surveillance. Trends Cancer.

[72] Yoshihama S., Roszik J., Downs I., Meissner T.B., Vijayan S., Chapuy B., Sidiq T., Shipp M.A., Lizee G.A., Kobayashi K.S. (2016). NLRC5/MHC class I transactivator is a target for immune evasion in cancer. Proc. Natl. Acad. Sci. USA.

[73] Taylor B.C., Balko J.M. (2022). Mechanisms of MHC-I Downregulation and Role in Immunotherapy Response. Front. Immunol..

[74] Nie Y., Yang G., Song Y., Zhao X., So C., Liao J., Wang L.D., Yang C.S. (2001). DNA hypermethylation is a mechanism for loss of expression of the HLA class I genes in human esophageal squamous cell carcinomas. Carcinogenesis.

[75] Nishida N., Aoki T., Morita M., Chishina H., Takita M., Ida H., Hagiwara S., Minami Y., Ueshima K., Kudo M. (2023). Non-Inflamed Tumor Microenvironment and Methylation/Downregulation of Antigen-Presenting Machineries in Cholangiocarcinoma. Cancers.

[76] Torii A., Harada A., Nakao A., Nonami T., Nakayama A., Ito M., Takagi H. (1992). Expression of HLA-DR in intrahepatic cholangiocarcinoma. Cancer.

[77] Bian J., Fu J., Wang X., Lee J., Brar G., Escorcia F.E., Cam M., Xie C. (2022). Characterization of Immunogenicity of Malignant Cells with Stemness in Intrahepatic Cholangiocarcinoma by Single-Cell RNA Sequencing. Stem Cells Int..

[78] Amengual J., Gonzalez-Sanchez E., Yáñez-Bartolome M., Sererols-Viñas L., Ravichandra A., Guiton C., Fuste N.P., Alay A., Hijazo-Pechero S., Martín-Mur B. (2025). NADPH oxidase 1/4 dual inhibition impairs transforming growth factor-beta protumorigenic effects in cholangiocarcinoma cancer-associated fibroblasts. Signal Transduct. Target. Ther..

[79] Chen Y., Ma L., He Q., Zhang S., Zhang C., Jia W. (2015). TGF-β1 expression is associated with invasion and metastasis of intrahepatic cholangiocarcinoma. Biol. Res..

[80] Liu Z., Weng S., Xu H., Wang L., Liu L., Zhang Y., Guo C., Dang Q., Xing Z., Lu T. (2021). Computational Recognition and Clinical Verification of TGF-β-Derived miRNA Signature With Potential Implications in Prognosis and Immunotherapy of Intrahepatic Cholangiocarcinoma. Front. Oncol..

[81] Meireson A., Devos M., Brochez L. (2020). IDO Expression in Cancer: Different Compartment, Different Functionality?. Front. Immunol..

[82] Cao L., Prithviraj P., Shrestha R., Sharma R., Anaka M., Bridle K.R., Kannourakis G., Crawford D.H., Jayachandran A. (2021). Prognostic Role of Immune Checkpoint Regulators in Cholangiocarcinoma: A Pilot Study. J. Clin. Med..

[83] Munir S., Larsen S.K., Iversen T.Z., Donia M., Klausen T.W., Svane I.M., Straten P.T., Andersen M.H. (2012). Natural CD4+ T-Cell Responses against Indoleamine 2,3-Dioxygenase. PLoS ONE.

[84] Van de Velde L.-A., Guo X.-Z.J., Barbaric L., Smith A.M., Oguin T.H., Thomas P.G., Murray P.J. (2016). Stress Kinase GCN2 Controls the Proliferative Fitness and Trafficking of Cytotoxic T Cells Independent of Environmental Amino Acid Sensing. Cell Rep..

[85] Mezrich J.D., Fechner J.H., Zhang X., Johnson B.P., Burlingham W.J., Bradfield C.A. (2010). An Interaction between Kynurenine and the Aryl Hydrocarbon Receptor Can Generate Regulatory T Cells. J. Immunol..

[86] Giacomantonio M.A., Vijayan V.V., Nair P.G., Pathak G.P., Kennedy B.E., Paulo J.A., McMillen T., Banerjee A., Kumar V., Sultonova M. (2026). Subversion of kynurenine-induced AHR activation in CD8 T cells by kynureninase-expressing antigen-presenting cells. Cell Rep..

[87] Plebanek M.P., Xue Y., Nguyen Y.-V., DeVito N.C., Wang X., Holtzhausen A., Beasley G.M., Theivanthiran B., Hanks B.A. (2024). A lactate-SREBP2 signaling axis drives tolerogenic dendritic cell maturation and promotes cancer progression. Sci. Immunol..

[88] Lu J.-C., Wu L.-L., Sun Y.-N., Huang X.-Y., Gao C., Guo X.-J., Zeng H.-Y., Qu X.-D., Chen Y., Wu D. (2024). Macro CD5L^+^ deteriorates CD8^+^T cells exhaustion and impairs combination of Gemcitabine-Oxaliplatin-Lenvatinib-anti-PD1 therapy in intrahepatic cholangiocarcinoma. Nat. Commun..

[89] Marabelle A., Le D.T., Ascierto P.A., Di Giacomo A.M., De Jesus-Acosta A., Delord J.-P., Geva R., Gottfried M., Penel N., Hansen A.R. (2020). Efficacy of Pembrolizumab in Patients with Noncolorectal High Microsatellite Instability/Mismatch Repair–Deficient Cancer: Results From the Phase II KEYNOTE-158 Study. J. Clin. Oncol..

[90] Poole R.M. (2014). Pembrolizumab: First Global Approval. Drugs.

[91] Freshwater T., Kondic A., Ahamadi M., Li C.H., de Greef R., de Alwis D., Stone J.A. (2017). Evaluation of dosing strategy for pembrolizumab for oncology indications. J. Immunother. Cancer.

[92] Cristescu R., Aurora-Garg D., Albright A., Xu L., Liu X.Q., Loboda A., Lang L., Jin F., Rubin E.H., Snyder A. (2022). Tumor mutational burden predicts the efficacy of pembrolizumab monotherapy: A pan-tumor retrospective analysis of participants with advanced solid tumors. J. Immunother. Cancer.

[93] Kim R.D., Chung V., Alese O.B., El-Rayes B.F., Li D., Al-Toubah T.E., Schell M.J., Zhou J.-M., Mahipal A., Kim B.H. (2020). A Phase 2 Multi-institutional Study of Nivolumab for Patients With Advanced Refractory Biliary Tract Cancer. JAMA Oncol..

[94] Gbolahan O., Hashemi-Sadraei N., O’neil B. (2019). Prolonged Response to Anti–PD-1 Antibody Therapy in Chemotherapy-Refractory Cholangiocarcinoma With High Tumor Mutational Burden. J. Natl. Compr. Cancer Netw..

[95] Di Federico A., Rizzo A., Ricci A.D., Frega G., Palloni A., Tavolari S., Brandi G. (2020). Nivolumab: An investigational agent for the treatment of biliary tract cancer. Expert Opin. Investig. Drugs.

[96] Oh D.-Y., Lee K.-H., Lee D.-W., Yoon J., Kim T.-Y., Bang J.-H., Nam A.-R., Oh K.-S., Kim J.-M., Lee Y. (2022). Gemcitabine and cisplatin plus durvalumab with or without tremelimumab in chemotherapy-naive patients with advanced biliary tract cancer: An open-label, single-centre, phase 2 study. Lancet Gastroenterol. Hepatol..

[97] Oh D.-Y., He A.R., Bouattour M., Okusaka T., Qin S., Chen L.-T., Kitano M., Lee C.-K., Kim J.W., Chen M.-H. (2024). Durvalumab or placebo plus gemcitabine and cisplatin in participants with advanced biliary tract cancer (TOPAZ-1): Updated overall survival from a randomised phase 3 study. Lancet Gastroenterol. Hepatol..

[98] Hack S.P., Zhu A.X. (2021). Atezolizumab: An investigational agent for the treatment of biliary tract cancer. Expert Opin. Investig. Drugs.

[99] Hack S.P., Verret W., Mulla S., Liu B., Wang Y., Macarulla T., Ren Z., El-Khoueiry A.B., Zhu A.X. (2021). IMbrave 151: A randomized phase II trial of atezolizumab combined with bevacizumab and chemotherapy in patients with advanced biliary tract cancer. Ther. Adv. Med. Oncol..

[100] Macarulla T., Ren Z., Chon H.J., Park J.O., Kim J.W., Pressiani T., Li D., Zhukova L., Zhu A.X., Chen M.-H. (2025). Atezolizumab Plus Chemotherapy With or Without Bevacizumab in Advanced Biliary Tract Cancer: Clinical and Biomarker Data From the Randomized Phase II IMbrave151 Trial. J. Clin. Oncol..

[101] Limousin W., Laurent-Puig P., Ziol M., Ganne-Carrié N., Nahon P., Ait-Omar A., Seror O., Sidali S., Campani C., Blanc P. (2023). Molecular-based targeted therapies in patients with hepatocellular carcinoma and hepato-cholangiocarcinoma refractory to atezolizumab/bevacizumab. J. Hepatol..

[102] Yarchoan M., Cope L., Ruggieri A.N., Anders R.A., Noonan A.M., Goff L.W., Goyal L., Lacy J., Li D., Patel A.K. (2021). Multicenter randomized phase II trial of atezolizumab with or without cobimetinib in biliary tract cancers. J. Clin. Investig..

[103] Klein O., Kee D., Nagrial A., Markman B., Underhill C., Michael M., Jackett L., Lum C., Behren A., Palmer J. (2020). Evaluation of Combination Nivolumab and Ipilimumab Immunotherapy in Patients With Advanced Biliary Tract Cancers. JAMA Oncol..

[104] Oh D.-Y., Ikeda M., Lee C.-K., Rojas C., Hsu C.-H., Kim J.W., Shen L., Furuse J., Park J.O., Borad M. (2024). Bintrafusp alfa and chemotherapy as first-line treatment in biliary tract cancer: A randomized phase 2/3 trial. Hepatology.

[105] Yoo C., Javle M.M., Mata H.V., de Braud F., Trojan J., Raoul J.-L., Kim J.W., Ueno M., Lee C.-K., Hijioka S. (2023). Phase 2 trial of bintrafusp alfa as second-line therapy for patients with locally advanced/metastatic biliary tract cancers. Hepatology.

[106] Teixeira A.F., Ten Dijke P., Zhu H.-J. (2020). On-Target Anti-TGF-β Therapies Are Not Succeeding in Clinical Cancer Treatments: What Are Remaining Challenges?. Front. Cell Dev. Biol..

[107] Kim B.-G., Malek E., Choi S.H., Ignatz-Hoover J.J., Driscoll J.J. (2021). Novel therapies emerging in oncology to target the TGF-β pathway. J. Hematol. Oncol..

[108] Biswas T., Gu X., Yang J., Ellies L.G., Sun L.-Z. (2014). Attenuation of TGF-β signaling supports tumor progression of a mesenchymal-like mammary tumor cell line in a syngeneic murine model. Cancer Lett..

[109] Burris H.A., Okusaka T., Vogel A., Lee M.A., Takahashi H., Breder V., Blanc J.-F., Li J., Bachini M., Żotkiewicz M. (2024). Durvalumab plus gemcitabine and cisplatin in advanced biliary tract cancer (TOPAZ-1): Patient-reported outcomes from a randomised, double-blind, placebo-controlled, phase 3 trial. Lancet Oncol..

[110] Reimann P., Mavroeidi I.-A., Burghofer J., Taghizadeh H., Webersinke G., Kasper S., Schreil G., Morariu D., Reichinger A., Baba H.A. (2024). Exploring the impact of durvalumab on biliary tract cancer: Insights from real-world clinical data. Cancer Immunol. Immunother..

[111] Kelley R.K., Ueno M., Yoo C., Finn R.S., Furuse J., Ren Z., Yau T., Klümpen H.-J., Ozaka M., Verslype C. (2023). Pembrolizumab in combination with gemcitabine and cisplatin compared with gemcitabine and cisplatin alone for patients with advanced biliary tract cancer (KEYNOTE-966): A randomised, double-blind, placebo-controlled, phase 3 trial. Lancet.

[112] Yoo C., Ueno M., Klümpen H.-J., Kelley R.K., Vogel A., Furuse J., Ren Z., Yau T., Chan S.L., Ozaka M. (2025). Health-related quality of life in participants with advanced biliary tract cancer from the randomized phase III KEYNOTE-966 study. J. Hepatol..

[113] Komuta M. (2022). Intrahepatic cholangiocarcinoma: Tumour heterogeneity and its clinical relevance. Clin. Mol. Hepatol..

[114] Makawita S., Lee S., Kong E., Kwong L.N., Abouelfetouh Z., De Armas A.D., Xiao L., Murugesan K., Danziger N., Pavlick D. (2024). Comprehensive Immunogenomic Profiling of *IDH1-*/*2*-Altered Cholangiocarcinoma. JCO Precis. Oncol..

[115] Xiang X., Liu Z., Zhang C., Li Z., Gao J., Zhang C., Cao Q., Cheng J., Liu H., Chen D. (2021). *IDH* Mutation Subgroup Status Associates with Intratumor Heterogeneity and the Tumor Microenvironment in Intrahepatic Cholangiocarcinoma. Adv. Sci..

[116] Pirozzi C.J., Yan H. (2021). The implications of IDH mutations for cancer development and therapy. Nat. Rev. Clin. Oncol..

[117] Wu J., Castro L.N.G., Battaglia S., El Farran C.A., D’antonio J.P., Miller T.E., Suvà M.L., Bernstein B.E. (2024). Evolving cell states and oncogenic drivers during the progression of IDH-mutant gliomas. Nat. Cancer.

[118] Ye D., Guan K.-L., Xiong Y. (2018). Metabolism, Activity, and Targeting of D- and L-2-Hydroxyglutarates. Trends Cancer.

[119] Nguyen M.K., Jelinek M., Singh A., Isett B., Myers E.S., Mullett S.J., Eisele Y., Beumer J.H., Parise R.A., Urban J. (2025). Clinical and translational study of ivosidenib plus nivolumab in advanced solid tumors harboring IDH1 mutations. Oncol..

[120] Huang X.-Y., Shi G.-M., Zheng Z.-T., Sun H.-C., Liang F., Ji Y., Chen Y., Yang G.-H., Hu Z.-Q., Lu J.-C. (2025). Anti-PD1 antibody toripalimab combined with lenvatinib, or GEMOX chemotherapy combined with lenvatinib as first-line therapy in patients with advanced intrahepatic cholangiocarcinoma: A randomized, open, two-cohort Phase 2 Study. BMC Med..

[121] Shi G.-M., Huang X.-Y., Wu D., Sun H.-C., Liang F., Ji Y., Chen Y., Yang G.-H., Lu J.-C., Meng X.-L. (2023). Toripalimab combined with lenvatinib and GEMOX is a promising regimen as first-line treatment for advanced intrahepatic cholangiocarcinoma: A single-center, single-arm, phase 2 study. Signal Transduct. Target. Ther..

[122] Shi G., Huang X., Li X., Liang F., Gao Q., Zhang D., Lu J., Ji Y., Hu Z., Chen Y. (2025). Conversion therapy of tislelizumab plus lenvatinib and GEMOX in unresectable locally advanced biliary tract cancer (ZSAB-TransGOLP): A multicentre, prospective, phase 2 study. Lancet Oncol..

[123] Garrido F., Perea F., Bernal M., Sánchez-Palencia A., Aptsiauri N., Ruiz-Cabello F. (2017). The Escape of Cancer from T Cell-Mediated Immune Surveillance: HLA Class I Loss and Tumor Tissue Architecture. Vaccines.

[124] Higashi M., Yonezawa S., Ho J.J.L., Tanaka S., Irimura T., Kim Y.S., Sato E. (1999). Expression of MUC1 and MUC2 mucin antigens in intrahepatic bile duct tumors: Its relationship with a new morphological classification of cholangiocarcinoma. Hepatology.

[125] Marks E.I., Yee N.S. (2015). Immunotherapeutic approaches in biliary tract carcinoma: Current status and emerging strategies. World J. Gastrointest. Oncol..

[126] Kaida M., Morita-Hoshi Y., Soeda A., Wakeda T., Yamaki Y., Kojima Y., Ueno H., Kondo S., Morizane C., Ikeda M. (2011). Phase 1 Trial of Wilms Tumor 1 (WT1) Peptide Vaccine and Gemcitabine Combination Therapy in Patients with Advanced Pancreatic or Biliary Tract Cancer. J. Immunother..

[127] Lepisto A.J., Moser A.J., Zeh H., Lee K., Bartlett D., McKolanis J.R., Geller B.A., Schmotzer A., Potter D.P., Whiteside T. (2008). A phase I/II study of a MUC1 peptide pulsed autologous dendritic cell vaccine as adjuvant therapy in patients with resected pancreatic and biliary tumors. Cancer Ther..

[128] Löffler M.W., Chandran P.A., Laske K., Schroeder C., Bonzheim I., Walzer M., Hilke F.J., Trautwein N., Kowalewski D.J., Schuster H. (2016). Personalized peptide vaccine-induced immune response associated with long-term survival of a metastatic cholangiocarcinoma patient. J. Hepatol..

[129] Maia A., Schuhmacher J., Nadalin S., Königsrainer A., Thiel K., Nelde A., Zinser R.S., Schroeder C., Mattern S., Singer S. (2025). Exceptional tumor-free survival of a patient with metastatic intrahepatic cholangiocarcinoma after surgery and personalized peptide vaccination: Revisiting a striking case. J. Immunother. Cancer.

[130] Shimizu K., Kotera Y., Aruga A., Takeshita N., Takasaki K., Yamamoto M. (2011). Clinical utilization of postoperative dendritic cell vaccine plus activated T-cell transfer in patients with intrahepatic cholangiocarcinoma. J. Hepato-Biliary-Pancreat. Sci..

[131] Rosenberg S.A., Restifo N.P., Yang J.C., Morgan R.A., Dudley M.E. (2008). Adoptive cell transfer: A clinical path to effective cancer immunotherapy. Nat. Rev. Cancer.

[132] Higuchi R., Yamamoto M., Hatori T., Shimizu K., Imai K., Takasaki K. (2006). Intrahepatic Cholangiocarcinoma with Lymph Node Metastasis Successfully Treated by Immunotherapy with CD3-Activated T Cells and Dendritic Cells After Surgery: Report of a Case. Surg. Today.

[133] Tran E., Turcotte S., Gros A., Robbins P.F., Lu Y.-C., Dudley M.E., Wunderlich J.R., Somerville R.P., Hogan K., Hinrichs C.S. (2014). Cancer Immunotherapy Based on Mutation-Specific CD4+ T Cells in a Patient with Epithelial Cancer. Science.

[134] Feng K., Liu Y., Guo Y., Qiu J., Wu Z., Dai H., Yang Q., Wang Y., Han W. (2017). Phase I study of chimeric antigen receptor modified T cells in treating HER2-positive advanced biliary tract cancers and pancreatic cancers. Protein Cell.

[135] Supimon K., Sangsuwannukul T., Sujjitjoon J., Phanthaphol N., Chieochansin T., Poungvarin N., Wongkham S., Junking M., Yenchitsomanus P.-T. (2021). Anti-mucin 1 chimeric antigen receptor T cells for adoptive T cell therapy of cholangiocarcinoma. Sci. Rep..

[136] Wongkajornsilp A., Somchitprasert T., Butraporn R., Wamanuttajinda V., Kasetsinsombat K., Huabprasert S., Maneechotesuwan K., Hongeng S. (2009). Human Cytokine-Induced Killer Cells Specifically Infiltrated and Retarded the Growth of the Inoculated Human Cholangiocarcinoma Cells in SCID Mice. Cancer Investig..

